# PFKP alleviates glucose starvation-induced metabolic stress in lung cancer cells via AMPK-ACC2 dependent fatty acid oxidation

**DOI:** 10.1038/s41421-022-00406-1

**Published:** 2022-05-31

**Authors:** Jiaqing Chen, Li Zou, Guang Lu, Oleg Grinchuk, Lei Fang, Derrick Sek Tong Ong, Reshma Taneja, Choon-Nam Ong, Han-Ming Shen

**Affiliations:** 1 NUS Graduate School Integrative Sciences and Engineering Programme (ISEP), National University of Singapore, Singapore, Singapore; 2 Department of Physiology, Yong Loo Lin School of Medicine, National University of Singapore, Singapore, Singapore; 3 Saw Swee Hock School of Public Health, National University of Singapore, Singapore, Singapore; 4 Zhongshan School of Medicine, Sun Yat-sen University, Guangzhou, China; 5 Jiangsu Key Laboratory of Molecular Medicine, Model Animal Research Center, Medical School of Nanjing University, Nanjing, Jiangsu China; 6 Faculty of Health Sciences, Ministry of Education Frontiers Science Center for Precision Oncology, University of Macau, Taipa, Macau China

**Keywords:** Cancer metabolism, Cell signalling

## Abstract

Cancer cells adopt metabolic reprogramming to promote cell survival under metabolic stress. A key regulator of cell metabolism is AMP-activated protein kinase (AMPK) which promotes catabolism while suppresses anabolism. However, the underlying mechanism of AMPK in handling metabolic stress in cancer remains to be fully understood. In this study, by performing a proteomics screening of AMPK-interacting proteins in non-small-cell lung cancer (NSCLC) cells, we discovered the platelet isoform of phosphofructokinase 1 (PFKP), a rate-limiting enzyme in glycolysis. Moreover, PFKP was found to be highly expressed in NSCLC patients associated with poor survival. We demonstrated that the interaction of PFKP and AMPK was greatly enhanced upon glucose starvation, a process regulated by PFKP-associated metabolites. Notably, the PFKP–AMPK interaction promoted mitochondrial recruitment of AMPK which subsequently phosphorylated acetyl-CoA carboxylase 2 (ACC2) to enhance long-chain fatty acid oxidation, a process helping maintenance of the energy and redox homeostasis and eventually promoting cancer cell survival under glucose starvation. Collectively, we revealed a critical non-glycolysis-related function of PFKP in regulating long-chain fatty acid oxidation via AMPK to alleviate glucose starvation-induced metabolic stress in NSCLC cells.

## Introduction

In cancer cells, metabolic reprogramming facilitates cell proliferation and survival under various circumstances^1–3^. Under nutrient-sufficient conditions, cancer cells accelerate anabolism by driving the metabolic flux forward; while under nutrient-restricted conditions, cancer cells utilize different sources of nutrients to fulfill anabolic needs and maintain energy and redox homeostasis. Glucose is crucial for energy production and anabolic metabolism in cancer^4–6^. The glycolysis pathway comprises a series of metabolic reactions, in which three irreversible reactions mediated by hexokinases, phosphofructokinases, and pyruvate kinases are rate-limiting steps. Given the importance of glucose in cancer metabolism, glucose deprivation induces metabolic stress, particularly the energy and redox stress^7–9^. To deal with metabolic stress, cancer cells utilize other nutrients, such as glutamine, fatty acids, branched-chain amino acids, and lactate, to fulfill metabolic needs^10–18^.

AMP-activated protein kinase (AMPK) is a key sensor and regulator of cell metabolism^19–21^. In mammalian cells, AMPK is a heterotrimeric complex, which consists of a catalytic α subunit and two regulatory β and γ subunits^19,20^. Under energy stress situations, AMPK senses the decreased ATP/AMP or ATP/ADP ratio directly through its γ subunit, which results in an allosteric activation of its kinase activity^22^. This allosteric change also facilitates the net phosphorylation on the AMPKα subunit at Thr172 by its upstream kinase liver kinase B1 (LKB1), which further enhances the kinase activity of AMPK^23^. Under glucose-deprived condition, AMPK also senses the level of glucose through the glycolysis enzyme aldolase and its substrate fructose-1,6-bisphosphate, leading to the LKB1-mediated phosphorylation of AMPK^24–26^. Upon activation via the mechanisms mentioned above, AMPK promotes catabolism, while inhibits anabolism to alleviate energy stress through multiple pathways^22,27^. So far, an array of proteins phosphorylated by AMPK are known to be involved in fatty acid metabolism, lipid and sterol synthesis, glycolysis, gluconeogenesis, autophagy, mitochondrial fission, etc^19,27–29^.

Besides the well-known upstream and downstream signaling pathways, AMPK is also regulated precisely in terms of its intracellular distribution and activation^30–32^. For instance, the localization of AMPK is known to be regulated by myristoylation of the β subunit which facilitates the association of AMPK with membrane structures and the activation of AMPK by LKB1^31,33,34^. Meanwhile, AMPK is also reported to interact with the pyruvate kinase M2, leading to the nuclear translocation of both proteins^35^. Moreover, the AMPK distributed in specific organelles is activated hierarchically depending on the severity of energy stress. Once glucose is deprived, the lysosome-associated AMPK is activated by LKB1 in an energy stress-independent manner^25,30^. Under modest energy stress, the increase of AMP activates the cytosolic AMPK; while under severe energy stress when the AMP level is high, the mitochondria-associated AMPK is activated^30^. As a result, the activated AMPK on lysosome or in cytosol targets a distinct subset of proteins, such as the metabolic enzyme acetyl-CoA carboxylase 1 (ACC1). In contrast, the mitochondria-localized ACC2 is phosphorylated by AMPK under severe energy stress^30^. At present, the precise mechanisms regulating specific organelle-associated AMPK remain to be fully understood.

In this study, to better understand the precise mechanism regulating AMPK and its function in metabolic reprogramming under metabolic stress, we conducted a proteomics screening of AMPK-interacting proteins in non-small-cell lung cancer (NSCLC) cells and identified a rate-limiting enzyme in glycolysis named the platelet isoform of phosphofructokinase 1 (PFKP). Bioinformatics analysis demonstrated that PFKP was highly expressed in NSCLC patients associated with poor survival. Of note, the PFKP–AMPK interaction was markedly enhanced upon glucose starvation (GS) due to the reduction of PFKP associated metabolites. Moreover, PFKP facilitated the mitochondrial recruitment of AMPK which subsequently phosphorylated ACC2 to promote long-chain fatty acid oxidation. Lastly, the enhanced consumption of long-chain fatty acids maintains energy and redox homeostasis to promote cancer cell survival under GS.

## Results

### PFKP is an AMPK-interacting protein under GS

**Fig. 1:**
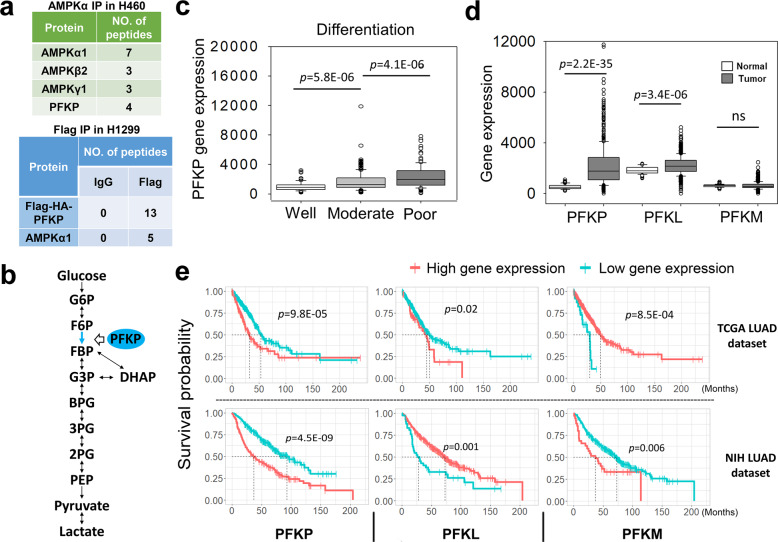
PFKP is an AMPK-interacting protein and highly expressed in lung adenocarcinoma. **a** Mass spectrometry analysis of interacting proteins using AMPKα or Flag-HA-PFKP as bait in NSCLC cells under GS for 4 h. **b** Diagram of glycolysis pathway and the step catalyzed by PFKP. G6P Glucose-6-Phosphate, F6P Fructose-6-Phosphate, FBP Fructose-1,6-bisphosphate, G3P Glyceraldehyde-3-Phosphate, DHAP Dihydroxyacetone-Phosphate, BPG 1,3-Bisphosphoglycerate, 3PG 3-Phosphoglycerate, 2PG 2-Phosphoglycerate, PEP Phosphoenolpyruvate. **c** Gene expression levels of PFKP in well, moderately, and poorly differentiated tumors from the NIH LUAD dataset. **d** Gene expression levels of *PFKP*, *PFKL*, and *PFKM* in tumor and adjacent normal tissue from the TCGA LUAD dataset. **e** Survival analysis of lung adenocarcinoma patients with high or low expression of *PFKP*, *PFKL*, and *PFKM* in the TCGA LUAD dataset (*n* = 501) and NIH LUAD dataset (*n* = 442). Data are shown as means ± SD with *n* indicating the number of patients in the datasets. *P* values by Mann–Whitney U test.

We have previously demonstrated the crucial role of AMPK in alleviating GS-induced oxidative stress in NSCLC^7^. To find out more proteins functionally associated with AMPK under metabolic stress, we performed a mass spectrometry-based proteomics screening of AMPK-interacting proteins in a NSCLC cell line H460 under GS and identified hundreds of targets. Since this assay was performed in cells under GS, we focused on glycolysis-related metabolic enzymes and found several such enzymes with high scores, including the rate-limiting enzyme pyruvate kinase M which has been reported to bind to AMPK under GS^35^. Notably, another rate-limiting enzyme PFKP was also discovered with a strong binding ability to AMPK (Fig. 1a and Supplementary Table S1). We then conducted another proteomics screening using Flag-HA tagged PFKP as the bait in another NSCLC cell line H1299. The results further confirmed PFKP as a binding partner of AMPK under GS (Fig. 1a and Supplementary Table S2).

### PFKP is highly expressed in lung adenocarcinoma

It is well known that PFKP is an isoform enzyme of phosphofructokinase 1 which catalyzes the rate-limiting step in glycolysis by converting fructose-6-phosphate (F6P) and ATP to fructose-1,6-bisphosphate (FBP) and ADP (Fig. 1b). In humans, there are three isoforms of phosphofructokinase 1 encoded by three different genes, i.e., the liver type (*PFKL*), muscle type (*PFKM*), and the platelet type (*PFKP*)^36,37^. Of note, PFKP is reported to be a highly expressed biomarker of poor prognosis in breast and lung cancer^38–40^.

To investigate the expression pattern of PFKP in NSCLC, we analyzed two clinical lung adenocarcinoma (LUAD) datasets. In the NIH LUAD dataset^41^, the PFKP expression was correlated with the differentiation level of tumor, the poorly differentiated LUAD with the highest level of PFKP (Fig. 1c). Meanwhile, higher level of PFKP was found in tumor tissue compared with adjacent normal tissue in the TCGA LUAD dataset (Fig. 1d). Moreover, survival analysis of the two datasets demonstrated that high expression of PFKP was associated with poor survival (Fig. 1e). In contrast, the results of PFKL and PFKM in survival analysis in the two datasets were not consistent (Fig. 1e). These results thus suggest that PFKP is highly expressed in LUAD with potential oncogenic functions.

### GS enhances the interaction between PFKP and AMPK

**Fig. 2:**
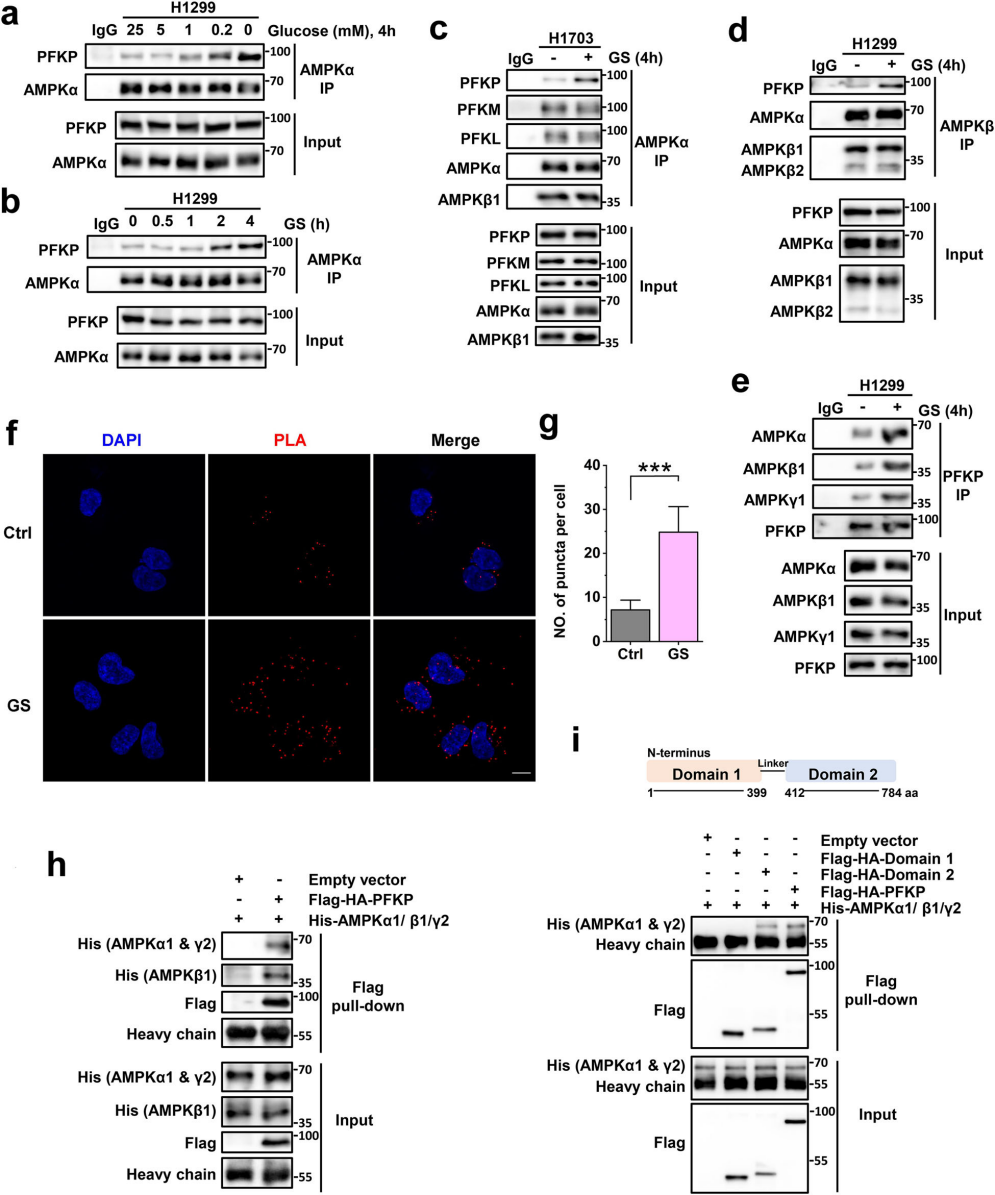
GS enhances the interaction between PFKP and AMPK. **a** Immunoprecipitation (IP) of AMPKα in H1299 cells treated with different levels of glucose. **b** AMPKα IP in H1299 cells treated with GS for different time points. **c**–**e** IP of AMPKα in H1703 (**c**), AMPKβ in H1299 (**d**), and PFKP in H1299 (**e**) cells under control (Ctrl) or GS. **f** Proximity ligation assay (PLA) of PFKP and AMPKβ1/2 in H1299 cells under control (Ctrl) or GS for 4 h. Scale bar, 10 μm. **g** Counts of PLA puncta in H1299 cells under control (*n* = 38) or GS (*n* = 44). Data are shown as means ± SD with *n* indicating the number of biological replicates. *P < 0.05, **P < 0.01, ***P < 0.001 by two-tailed Student’s *t*-test. **h**, **i** In vitro protein–protein interaction assay of purified PFKP (**h**) or PFKP domains (**i**) with AMPK. Flag-HA tagged PFKP and PFKP domains were purified by Flag IP and then incubated with His-tagged AMPKα1/β1/γ2 for 1 h at room temperature.

To investigate the interaction between PFKP and AMPK, we cultured the cells in medium with different levels of glucose and observed that their interaction was strongly enhanced under low glucose conditions (Fig. 2a). Next, upon exposure to GS, the PFKP-AMPK interaction was enhanced in a time-dependent pattern (Fig. 2b). To further confirm the interaction, we performed immunoprecipitations of AMPKα, AMPKβ, and PFKP in three different NSCLC cell lines (H1299, H1703, and H358) respectively. It was observed that GS greatly enhanced the interaction between PFKP and AMPK comparing to that under control condition (Fig. 2c–e and Supplementary Fig. S1a). In contrast to PFKP, the interaction of PFKL or PFKM with AMPK was not affected by GS (Fig. 2c). Moreover, the proximity ligation assay of PFKP and AMPK also demonstrated their enhanced interaction under GS in H1299 cells (Fig. 2f, g). To further verify the observed results, we performed in vitro protein-protein interaction assay using purified PFKP and AMPK and observed the interaction between these two proteins (Fig. 2h and Supplementary Fig. S1b, c).

In humans, AMPK α and β subunits have two isoforms respectively and the γ subunit has three isoforms, and the expressions of these isoforms vary among cancer cells^22,27^. To evaluate the influence of different isoforms on the PFKP-AMPK interaction, we conducted immunoprecipitations of AMPKα isoforms respectively, as well as in vitro protein-protein interaction assays using different subunit isoforms of AMPK. We observed that PFKP bound to isoform complexes of AMPK with similar affinity (Fig. 2e, h and Supplementary Fig. S1c, d). Additionally, PFKP has an N-terminal catalytic domain (Domain 1) and a C-terminal regulatory domain (Domain 2)^42^. To monitor the interacting region of PFKP, we purified the two domains of PFKP and found that the domain 2 interacted with AMPK (Fig. 2i), indicating that the regulatory domain of PFKP is responsible for its interaction with AMPK. Collectively, these data suggest that GS enhances the interaction between PFKP and AMPK.

### The PFKP–AMPK interaction is independent of AMPK activity but regulated by PFKP-associated metabolites

**Fig. 3:**
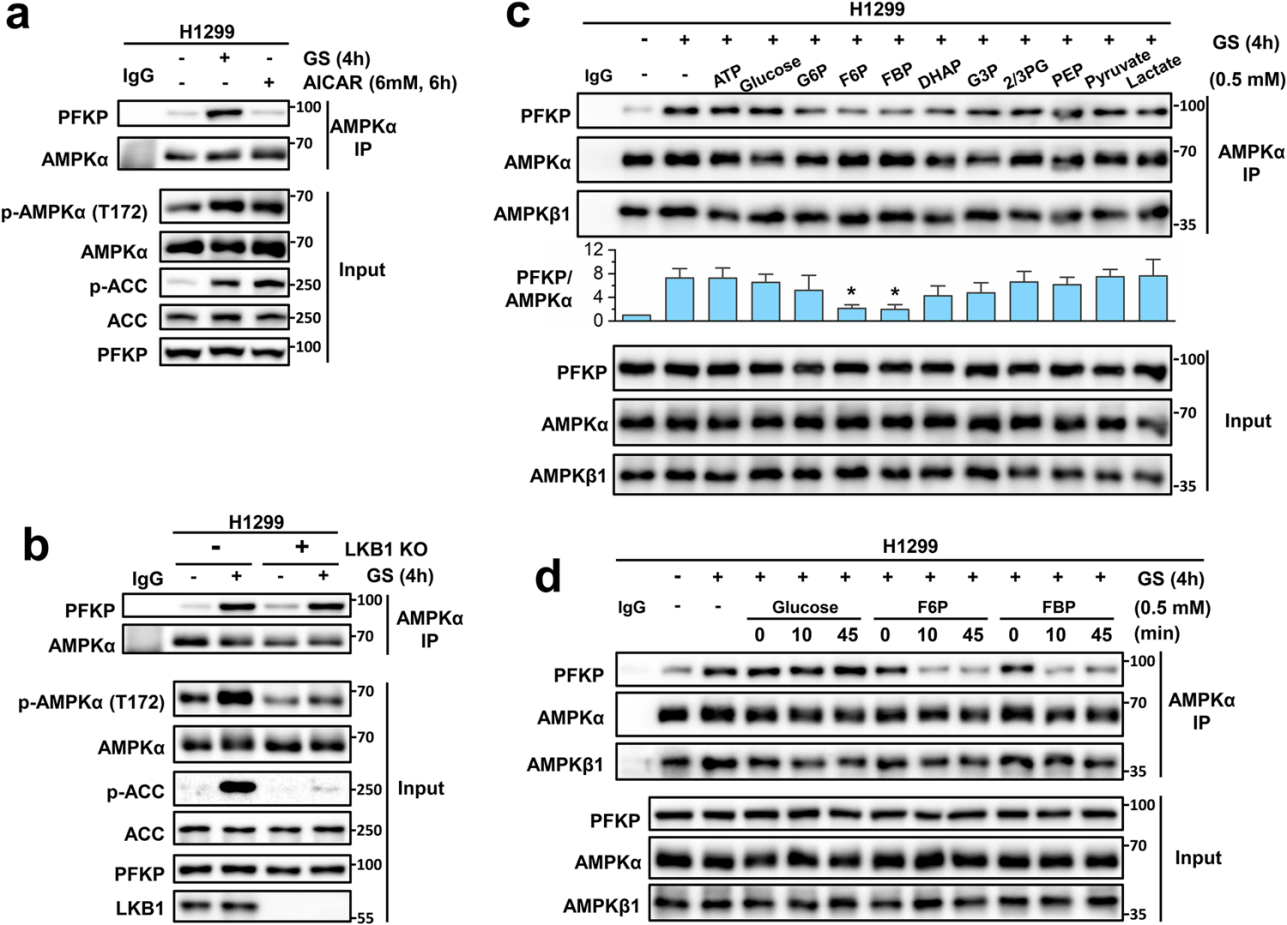
The PFKP-AMPK interaction is independent of AMPK activity but regulated by PFKP-associated metabolites. **a** AMPKα IP in H1299 cells under control, GS, or AICAR treatment. **b** AMPKα IP in H1299 cells with or without LKB1 under control or GS. **c** AMPKα IP in H1299 cells under control or GS. The indicated metabolites were incubated with the cell lysate at room temperature for 30 min followed by IP. The difference between GS samples with or without metabolites were compared. **d** AMPKα IP in H1299 cells under control or GS. Glucose, F6P, and FBP were incubated with the cell lysate at room temperature for 0, 10, or 45 min followed by IP. Data are shown as means ± SD with three biological replicates. *P < 0.05, **P < 0.01, ***P < 0.001 by two-tailed Student’s *t*-test. G6P Glucose-6-Phosphate, F6P Fructose-6-Phosphate, FBP Fructose-1,6-bisphosphate, DHAP Dihydroxyacetone-Phosphate, G3P Glyceraldehyde-3-Phosphate, 2/3PG 2/3-Phosphoglycerate, PEP Phosphoenolpyruvate.

To understand the underlying mechanism of the enhanced PFKP-AMPK interaction under GS, we treated the H1299 cells with 5-aminoimidazole-4-carboxamide ribonucleotide (AICAR), a membrane-permeable analog of AMP, to activate AMPK^30^. It was observed that the PFKP–AMPK interaction was not enhanced by AICAR, although this treatment induced AMPK activation comparable to that under GS, as indicated by the increased p-AMPK and p-ACC level (Fig. 3a). Here, though AICAR has other AMPK-independent effects and more specific agonists such as MK-8722^43^ or Compound 13^44^ are recommended, the negative results in Fig. 3a are not affected by those off-target effects of AICAR. Meanwhile, since LKB1 is the main kinase responsible for AMPK phosphorylation and activation under GS^7^, we established the LKB1 knock-out (KO) H1299 cells using CRISPR-Cas9 technique and monitored the PFKP-AMPK interaction. We found that in LKB1 KO cells GS failed to activate AMPK, but the PFKP-AMPK interaction induced by GS was unaffected (Fig. 3b). Collectively, these data indicate that the PFKP-AMPK interaction is independent of the AMPK kinase activity.

Since GS inhibits glycolysis and causes the reduction of metabolites produced in the glycolysis process^25,45^, we suspected that the PFKP-AMPK interaction may be associated with reduced glycolytic metabolites. To test this hypothesis, we added back the respective metabolites and ATP into the GS-treated cell lysates of H1299 and then examined the PFKP-AMPK interaction. The immunoprecipitation results demonstrated that the PFKP-AMPK interaction was suppressed by PFKP-associated metabolites, F6P and FBP, but not ATP or other glycolytic metabolites (Fig. 3c). Moreover, we incubated the lysates with glucose, F6P, or FBP for different durations at room temperature to see how rapidly the interaction can be reduced. This in vitro experiment showed that the effects of F6P and FBP on PFKP-AMPK interaction were obvious with 10 min incubation (Fig. 3d). Hence, these data indicate that the PFKP-AMPK interaction is induced by the reduction of PFKP-associated metabolites under GS.

### PFKP promotes mitochondrial recruitment of AMPK

**Fig. 4:**
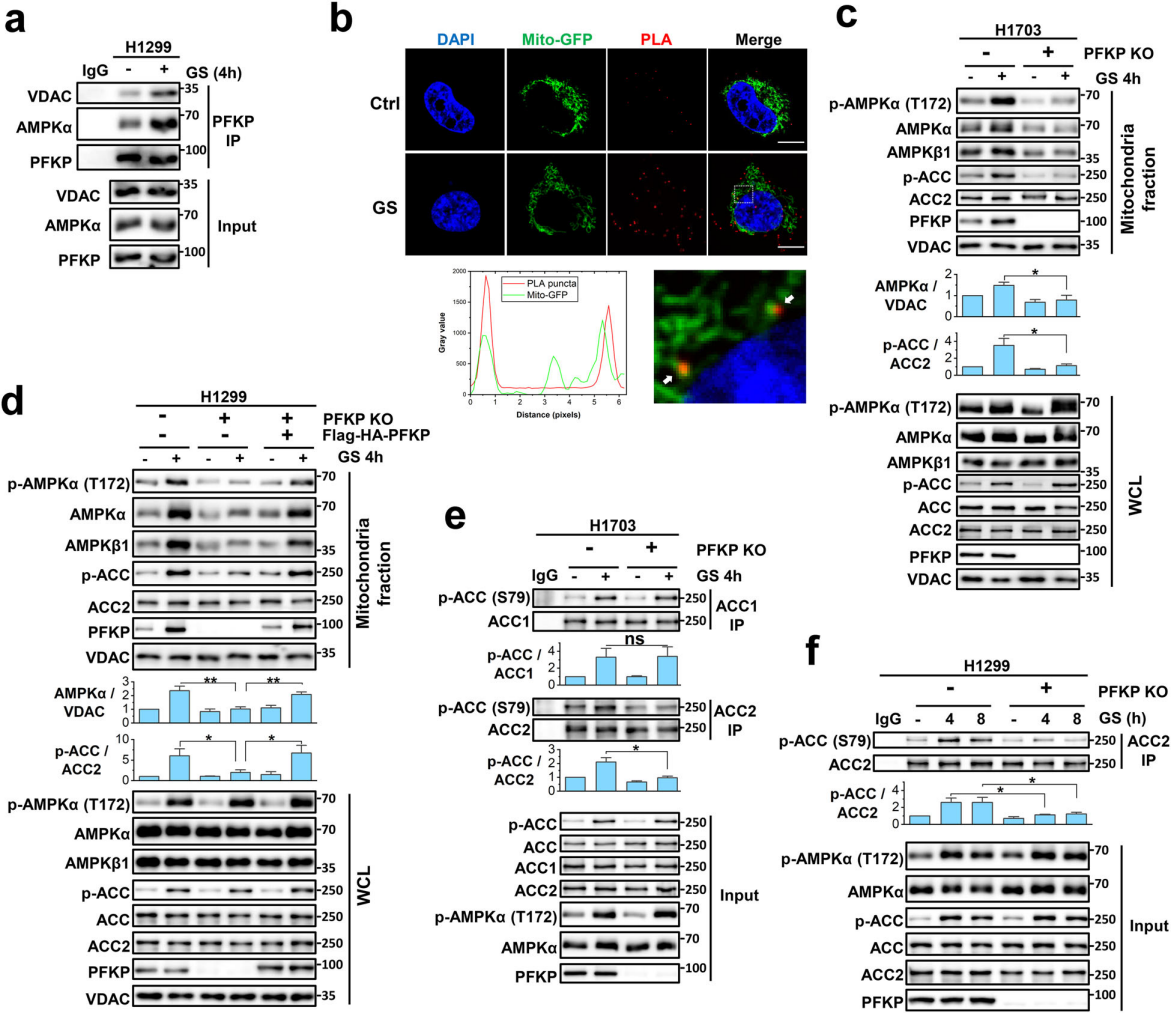
PFKP promotes mitochondrial recruitment of AMPK and enhances the phosphorylation of ACC2. **a** PFKP IP in H1299 cells under control or GS. **b** Immunofluorescence analysis of mito-GFP and PLA puncta (interacted PFKP and AMPK) in H1299 cells under control (Ctrl) or GS for 4 h. The gray values of green and red signals between two the arrows were plotted. Scale bar, 10 μm. **c**, **d** Mitochondria fraction analysis of H1703 (**c**) and H1299 (**d**) cells under control or GS. **e** IP of ACC1 and ACC2 in H1703 cells under control or GS. **f** ACC2 IP in H1299 cells under control or treated with GS for 4 h or 8 h. Data are shown as means ± SD with three biological replicates. ns, no significance; *P < 0.05, **P < 0.01, ***P < 0.001 by two-tailed Student’s *t*-test.

It has been reported that PFKP can regulate the transcriptional activity of Yes-associated protein/Transcriptional coactivator with PDZ-binding motif (YAP/TAZ)^46^ and the activation of phosphoinositide 3-kinase (PI3K)^47^ through direct binding with these proteins. Moreover, PFKP is known to be associated with mitochondria via binding to the voltage-dependent anion channel (VDAC)^48^. Consistently, we observed the interaction between PFKP and VDAC and found that their interaction was enhanced upon GS in H1299 cells (Fig. 4a). Besides, the enhanced association of PFKP with mitochondria upon GS was confirmed in the co-localization analysis of mito-GFP and PFKP in H1299 cells by Immunofluorescence staining (Supplementary Fig. S2a). Next, we investigated whether PFKP can interact with AMPK on mitochondria and verified their interaction in both cytosol and mitochondria fraction of H1703 cells (Supplementary Fig. S2b, c), suggesting that AMPK may associate with mitochondria via PFKP.

Thereafter, we checked whether the interacted PFKP-AMPK is associated with mitochondria upon GS using mito-GFP as a mitochondrial marker and the PLA signal as an indication of PFKP-AMPK interaction. Indeed, we found that GS enhanced the localization of PFKP-AMPK at mitochondria in H1299 cells (Fig. 4b). This finding was also verified by the co-localization analysis of mito-GFP with AMPK, as well as the protein levels of AMPK and PFKP in the mitochondria fraction (Supplementary Fig. S2d, e). We also established the PFKP KO cells using CRISPR-Cas9 technique and found that the co-localization of AMPK with mitochondria was abolished by the depletion of PFKP in H1299 cells (Supplementary Fig. S2d). Meanwhile, to understand the mechanisms that regulate the mitochondrial association of PFKP and AMPK, we knocked down VDAC in H1703 cells and found that the knock-down of VDAC diminished the increased protein levels of both PFKP and AMPK in the mitochondrial fraction under GS (Supplementary Fig. S2f). Collectively, these data suggest that PFKP may recruit AMPK onto the mitochondria via its interaction with VDAC and AMPK.

To further investigate the role of PFKP in regulating AMPK, we examined the level and activity of mitochondria-associated AMPK. First, we found that GS significantly enhanced the PFKP and AMPK level in the mitochondrial fraction in the wild-type H1703 and H358 cells (Fig. 4c and Supplementary Fig. S3a), suggesting that GS promotes the mitochondrial translocation of PFKP and AMPK. Second, we observed that GS failed to increase the AMPK level in the mitochondrial fraction of PFKP KO cells (Fig. 4c and Supplementary Fig. S3a), indicating that the GS-induced mitochondrial translocation of AMPK is PFKP-dependent. Meanwhile, we noticed that though the total levels of p-AMPK in the whole-cell lysis under GS were comparable, the p-AMPK level in the mitochondrial fraction of PFKP KO cells was much lower than that in wild-type cells (Fig. 4c and Supplementary Fig. S3a), suggesting that the suppressed mitochondrial recruitment of AMPK in PFKP KO cells leads to the reduction of p-AMPK on the mitochondria under GS. Third, to confirm the above observations, we established the stable cell lines with reconstitution of Flag-HA tagged PFKP in H1299 PFKP KO cells and found that re-expression of PFKP largely restored the AMPK and p-AMPK level in the mitochondrial fraction under GS (Fig. 4d). Lastly, since it has been reported that AMPK myristoylation promotes its mitochondrial distribution and activation^31,33,34^, we examined the level of myristoylated AMPKβ and observed no influence of PFKP on AMPKβ myristoylation in H1299 cells (Supplementary Fig. S3b), suggesting that the effects of PFKP on mitochondrial recruitment of AMPK is not relevant to myristoylation. Taken together, data from this part of the study clearly demonstrate that PFKP facilitates the mitochondrial recruitment of AMPK and enhances the level of activated AMPK on mitochondria under GS.

### PFKP promotes ACC2 phosphorylation via mitochondrial AMPK

Upon the mitochondrial recruitment of AMPK by PFKP, the mitochondria-localized ACC2 is expected to be targeted and phosphorylated by AMPK^17,49^. Indeed, we observed that GS markedly enhanced p-ACC level in the mitochondrial fraction in various lung cancer cell lines, and such changes were found to be PFKP-dependent (Fig. 4c, d and Supplementary Fig. S3a). As only ACC2 is known to be associated with mitochondria^17^, the enhanced p-ACC level in the mitochondrial fraction indicates the increased phosphorylation of ACC2, suggesting that the PFKP-mediated mitochondrial recruitment of AMPK contributes to the increased ACC2 phosphorylation.

To further validate this finding, we pulled down ACC1 and ACC2 respectively from the whole cell lysate of H1703 and found that the phosphorylation of cytosol-localized ACC1 was not affected by the deletion of PFKP under GS, while the level of phosphorylated ACC2 was significantly reduced (Fig. 4e and Supplementary Fig. S3c). This set of data further support the notion that PFKP plays an important role in promoting ACC2 phosphorylation via the mitochondrial recruitment of AMPK under GS. Moreover, we also noticed that the effect of PFKP in regulating ACC2 phosphorylation was persistent (last up to 8 h) in H1299 cells (Fig. 4f), suggesting that PFKP may have continuous influence on the enzyme activity of ACC2 under GS. Taken together, these data demonstrate that the recruited AMPK by PFKP enhances ACC2 phosphorylation on the mitochondria upon GS.

### PFKP promotes long-chain fatty acid oxidation via ACC2 to maintain energy and redox homeostasis

**Fig. 5:**
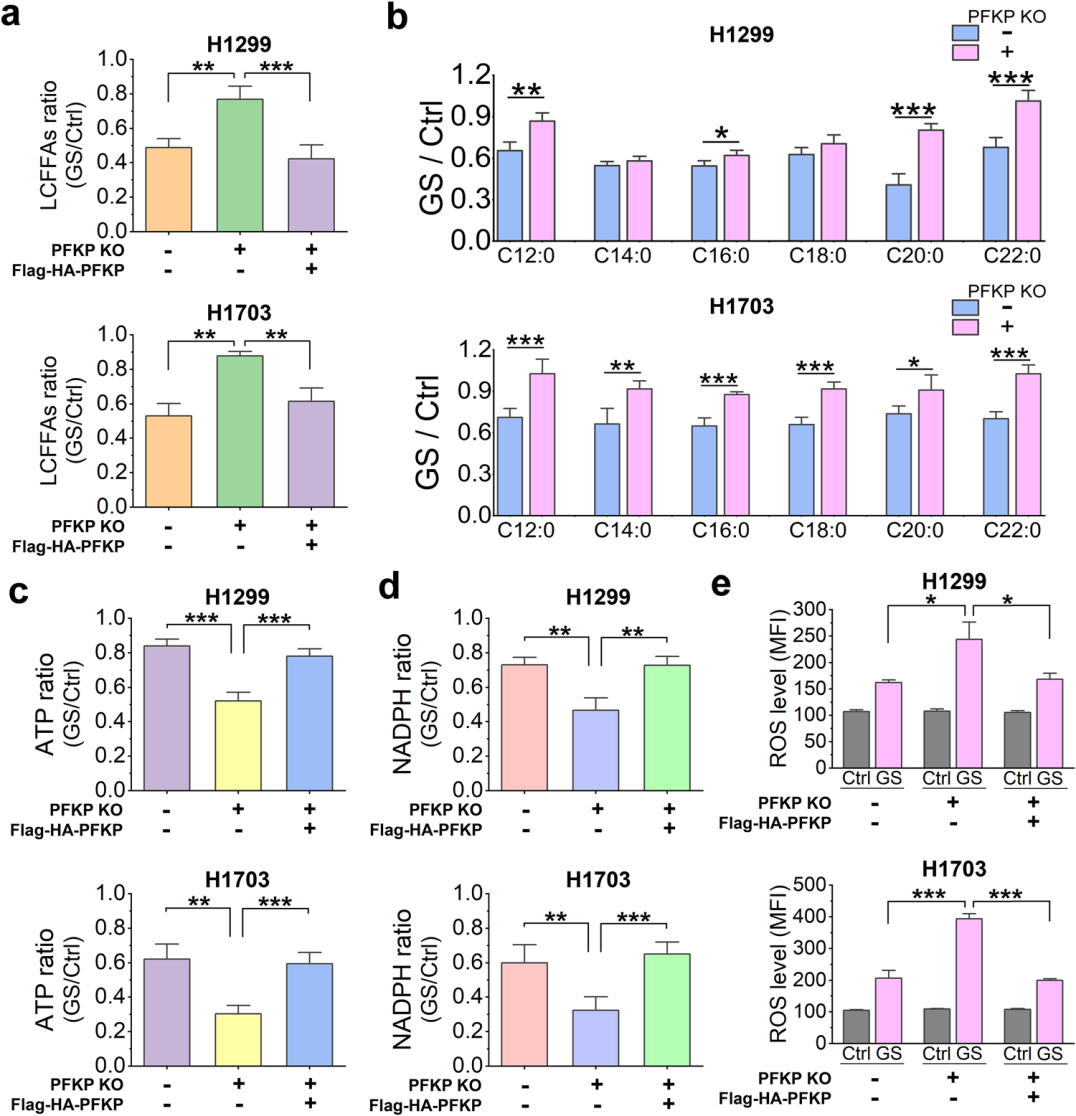
PFKP promotes long-chain fatty acid oxidation via ACC2 to maintain energy and redox homeostasis. **a** Relative level of long-chain free fatty acids (LCFFAs) in H1299 (*n* = 4) or H1703 (*n* = 4) cells under GS for 14 h over that in control. **b** Relative concentration of lauric acid (C12:0), myristic acid (C14:0), palmitic acid (C16:0), stearic acid (C18:0), arachidic acid (C20:0), and behenic acid (C22:0) in H1299 (*n* = 4) or H1703 (*n* = 5) cells under GS for 14 h over that in control. **c–e** Relative ATP (**c**, *n* = 4), NADPH (**d**, *n* = 4), and ROS level (**e**, *n* = 6) in H1299 or H1703 cells under GS for 14 h over that in control (Ctrl). Data are shown as means ± SD with *n* indicating the number of biological replicates. *P < 0.05, **P < 0.01, ***P < 0.001 by two-tailed Student’s *t*-test.

It is known that the phosphorylated ACC1 inhibits fatty acid synthesis while the phosphorylated ACC2 promotes fatty acid oxidation through regulating the long-chain fatty acids transporter carnitine palmitoyltransferase 1 (CPT1)^17^. Thus, it is possible that PFKP may indirectly regulate long-chain fatty acid oxidation via the AMPK-ACC2 signaling axis at mitochondria under GS. To test this possibility, we measured the concentration of long-chain free fatty acids by a kit targeting free fatty acids (C8 and longer) and calculated the ratio of the concentrations in H1299, H1703, and H358 cells under GS over that under control. We found that GS markedly reduced the level of long-chain free fatty acids in cells expressing PFKP, but no such obvious reduction was found in PFKP KO cells (Fig. 5a and Supplementary Fig. S4), suggesting that the GS-induced reduction of long-chain fatty acids is largely dependent on PFKP. Moreover, these changes were applicable to several types of long-chain fatty acids quantified by mass spectrometry in H1299 and H1703 cells (Fig. 5b). Since ACC1 phosphorylation was not relevant to PFKP (Fig. 4e), suggesting that the inhibition of fatty acid synthesis under GS is comparable between those cell lines. Therefore, the PFKP-dependent reduction of long-chain fatty acids demonstrate that PFKP can promote the long-chain fatty acid oxidation via the AMPK-ACC2 signaling pathway under GS.

Long-chain fatty acids are an alternative source of energy through fatty acid oxidation in mitochondria, especially under metabolic stress conditions^18,50^. In addition, the fatty acid oxidation also facilitates the production of the reduced form of nicotinamide adenine dinucleotide phosphate (NADPH), a key antioxidant in maintaining redox homeostasis^17,51,52^. Hence, we measured the intracellular concentrations of ATP, NADPH, and reactive oxygen species (ROS) in our cell models of H1299 and H1703. We found that deletion of PFKP caused significant reduction of ATP and NADPH level (Fig. 5c, d), accompanied by higher level of ROS (Fig. 5e). Meanwhile, reconstitution of PFKP in the PFKP KO cells restored the ATP, NADPH, and ROS level (Fig. 5c–e). Thus, our results clearly indicate that PFKP plays an important role in the production of ATP and NADPH under GS to maintain energy and redox homeostasis in the NSCLC cells.

### The regulatory effects of PFKP are independent of its enzyme activity or the catalytic domain, but dependent on AMPK

As a metabolic enzyme, we wondered whether the enzyme activity of PFKP was critical for its regulations of AMPK/ACC2 pathway and the long-chain fatty acid oxidation. We treated the H1703 cells with an inhibitor 2,5-Anhydro-D-glucitol-1,6-diphosphate^53^ and the results demonstrated that the inhibition of PFKP enzyme activity had no influence on the mitochondrial AMPK, ACC2 phosphorylation, long-chain free fatty acids level, and ROS level under GS (Supplementary Fig. S5). Moreover, we also investigated the catalytic domain (domain 1) which cannot interact with AMPK (Fig. 2i). As expected, the mitochondria fraction data clearly demonstrated that the re-expressed domain 1 in H1703 PFKP KO cells could translocate to mitochondria upon GS but it failed to recruit AMPK and enhance ACC2 phosphorylation (Supplementary Fig. S6a). All these results suggest that the regulatory effects of PFKP are independent of its enzyme activity or the catalytic domain.

On the other hand, we knocked down AMPKα in H1703 cells simultaneously to test whether the effects of PFKP was dependent on AMPK. The results clearly showed that AMPK knock-down could restore the reduced level of long-chain free fatty acids and ROS under GS (Supplementary Fig. S6b), suggesting that the regulatory activity of PFKP in long-chain fatty acids oxidation and redox balance is AMPK-dependent.

### PFKP promotes NSCLC cell survival under GS via long-chain fatty acid oxidation

**Fig. 6:**
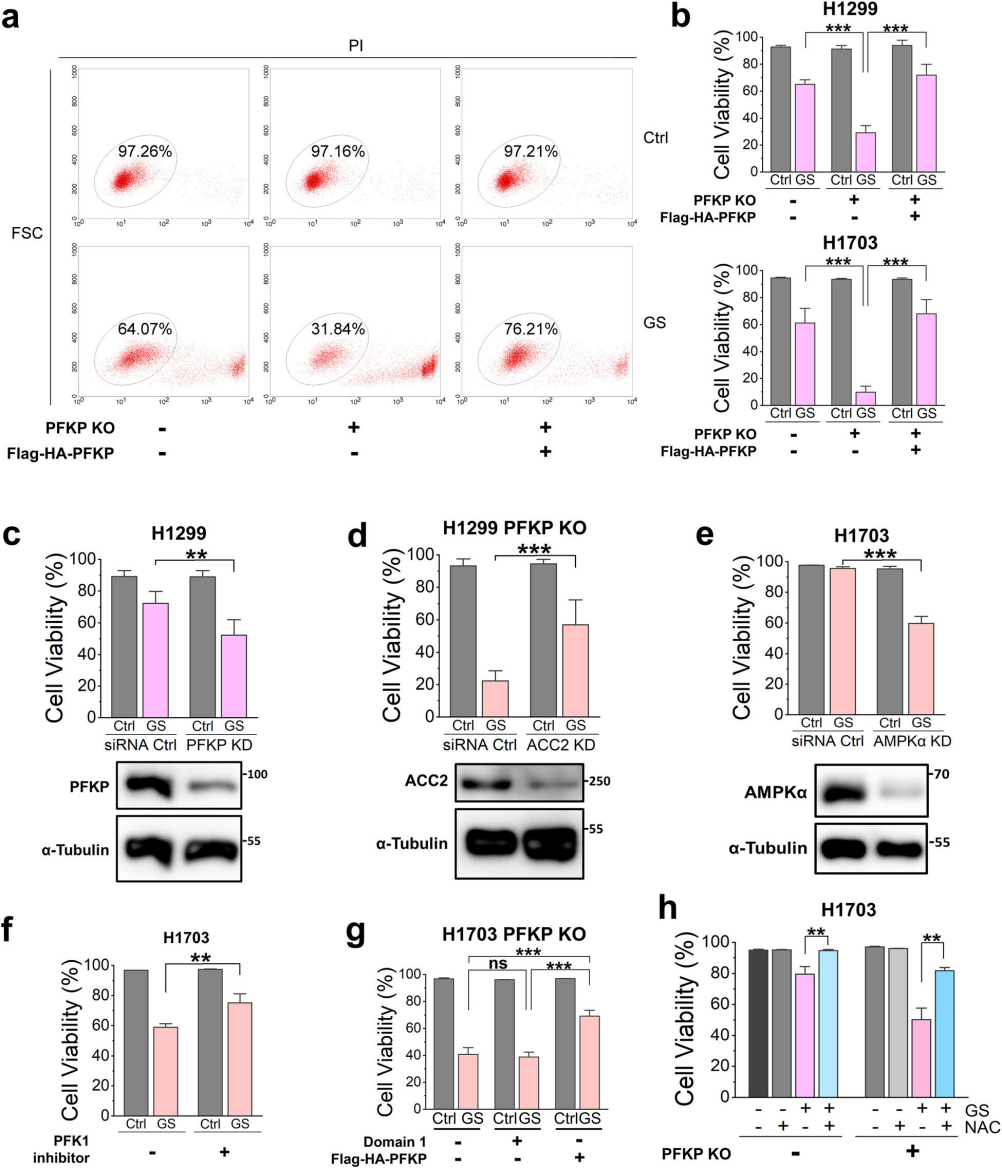
PFKP promotes NSCLC cell survival under GS via long-chain fatty acid oxidation. **a** Propidium iodide (PI) exclusion test of H1299 cells under control (Ctrl) or GS for 48 h. FSC: forward scatter. **b** Cell viability of H1299 (*n* = 9) or H1703 cells (*n* = 8) under control (Ctrl) or GS for 48 h. **c** Cell viability of H1299 with PFKP knock-down (KD) (*n* = 5) under control (Ctrl) or GS for 48 h. **d** Cell viability of H1299 KO cells with ACC2 KD (*n* = 9) under control (Ctrl) or GS for 48 h. **e** Cell viability of H1703 cells with AMPKα KD (*n* = 4) under control (Ctrl) or GS for 8 h. **f** Cell viability of H1703 cells treated with 1 mM PFKP inhibitor 2,5-Anhydro-D-glucitol-1,6-diphosphate (*n* = 4) under control (Ctrl) or GS for 24 h. **g** Cell viability of H1703 PFKP KO cells transfected with empty vector, Domain 1, or Flag-HA tagged PFKP (*n* = 4) under control (Ctrl) or GS for 24 h. **h** Cell viability of H1703 treated with 5 mM N-acetylcysteine (NAC) (*n* = 4) under control (Ctrl) or GS for 24 h. Data are shown as means ± SD with *n* indicating the number of biological replicates. *P < 0.05, **P < 0.01, ***P < 0.001 by two-tailed Student’s *t*-test.

Since the intracellular energy and redox homeostasis is critical for cell proliferation and survival, we suspected that the PFKP-mediated long-chain fatty acid oxidation can promote cancer cell survival under GS. We found that deletion of PFKP markedly sensitized H1299 and H1703 cells to GS-induced cell death, which were alleviated by the reconstitution of PFKP (Fig. 6a, b and Supplementary Fig. S7). Similarly, transient knock-down of PFKP in H1299 cells showed a similar trend to GS-induced cell death (Fig. 6c).

Moreover, the ACC2 knock-down in H1299 PFKP KO cells, mimicking the inhibitory phosphorylation of ACC2, significantly enhanced cell viability under GS (Fig. 6d), suggesting that the inhibition of ACC2 can indeed promote cancer cell survival under GS.

Similarly, knock-down of AMPK in H1703 cells could result in more cell death under GS (Fig. 6e), indicating that the regulatory effects of PFKP on cell death is AMPK-dependent. On the contrary, either inhibition of PFKP’s enzyme activity or re-expression of its domain 1 could not recue GS-induced cell death in H1703 cells (Fig. 6f, g), demonstrating that the effect of PFKP on GS-induced cell death is independent of its enzyme activity or the catalytic domain.

Previously, we reported that GS induces ROS-dependent cell death^7^. Consistently, we found that the GS-induced cell death could be partially rescued by the antioxidant N-acetylcysteine (NAC) in H1703 cells (Fig. 6h), indicating that the elevated intracellular ROS are the main cause of GS-induced cell death. These data collectively demonstrate that PFKP plays an important role in alleviating GS-induced metabolic stress and promoting cell survival under GS via enhancing long-chain fatty acid oxidation by the AMPK-ACC2 signaling pathway in NSCLC cells.

## Discussion

**Fig. 7:**
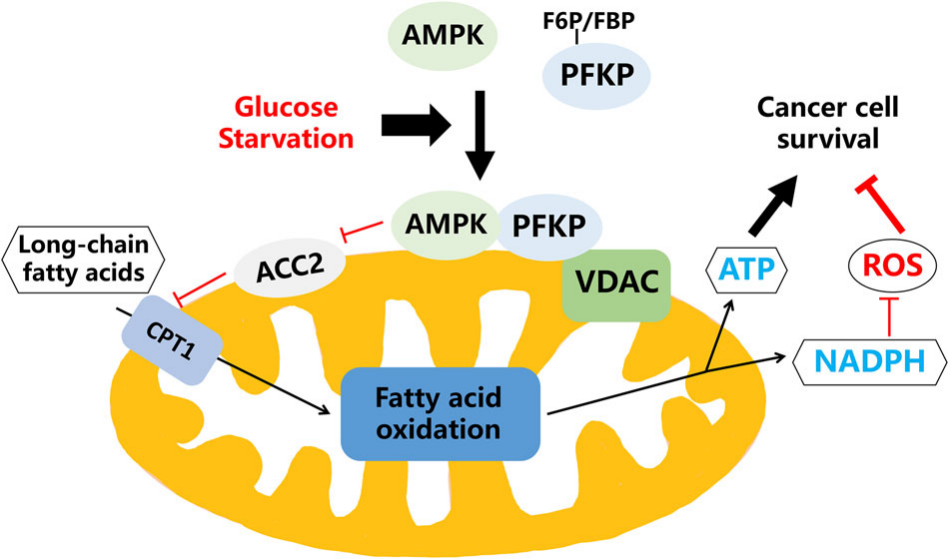
Schematic model of the PFKP-AMPK-ACC2-mediated long-chain fatty acid oxidation which promotes cancer cell survival under GS. PFKP mediates the recruitment and activation of mitochondrial AMPK under GS, which results in enhanced mitochondrial ACC2 phosphorylation and facilitates long-chain fatty acid oxidation to maintain metabolic homeostasis, leading to the promotion of cancer cell survival.

To better understand the regulation mechanism of AMPK and decipher its function in metabolic stress in cancer, this study discovers a novel function of PFKP, a rate-limiting enzyme in glycolysis, in regulating cellular energy and redox homeostasis with AMPK (Fig. 7). Upon GS, PFKP senses the decrease of its glycolytic metabolites (F6P and FBP), enhances the mitochondrial recruitment of AMPK, and promotes AMPK-mediated ACC2 phosphorylation and long-chain fatty acid oxidation. Eventually, the PFKP/AMPK/ACC2-mediated long-chain fatty acid oxidation facilitates ATP and NDAPH production to alleviate GS-induced metabolic stress, maintain cellular energy and redox homeostasis, and thereafter promote cancer cell survival.

Though PFKP is known as a glycolytic enzyme, it has been reported to promote cancer cell proliferation through its non-glycolysis-related functions in regulating YAP/TAZ and PI3K activity^46,47^. In this study, PFKP is found to be highly expressed in NSCLC patients associated with poor survival (Fig. 1c–e). More importantly, we discover the interaction of PFKP with AMPK under GS. Indeed, another glycolysis enzyme aldolase has a similar pattern of action: aldolase senses the level of FBP and triggers AMPK activation on lysosome^24–26^. Similar to aldolase, PFKP senses the associated metabolites and mediates its interaction with AMPK upon GS. Unlike aldolase, the interaction between PFKP and AMPK does not affect the overall activation of AMPK, as indicated by the p-AMPK and p-ACC level in the whole cell lysate (Fig. 4c, d and Supplementary Fig. S3a). However, PFKP influences the mitochondrial recruitment of AMPK (Fig. 4c, d and Supplementary Fig. S3a) and promotes NSCLC cell survival under GS (Fig. 6a–c). Besides, these effects of PFKP are independent of its enzyme activity (Supplementary Fig. S5) but largely dependent on AMPK (Supplementary Fig. S6). All these findings demonstrate that PFKP functions not only as a glycolysis enzyme, but more importantly as a potential oncogenic regulator in cancer.

Data from our study also expand the scope of AMPK’s function in response to metabolic stress. At present, the exact role of AMPK in cancer remains elusive. Under nutrient-sufficient conditions, AMPK is considered as a tumor suppressor since it inhibits anabolic metabolism^54–57^. Under metabolic stress conditions, however, there are evidences demonstrating that AMPK can promote cancer cell survival by maintaining energy and redox homeostasis^7–9^, promoting glucose metabolism and fatty acid oxidation^8,18,58^, upregulating lysosome generation^59^, and suppressing nucleotide synthesis^60^. In this study, we provide convincing evidence showing that AMPK is capable of promoting long-chain fatty acid oxidation via the PFKP-AMPK-ACC2 signaling pathway under GS, leading to the promoted ATP and NADPH production and cell survival in NSCLC cells. Therefore, data from our study provide a new line of evidence to indicate the oncogenic function of AMPK in NSCLC, a process implicating PFKP.

One important metabolic characteristic of cancer cells is their ability to utilize different nutrients to support cell proliferation and survival. It has been reported that glucose^4^, glutamine^15^, fatty acids^17,61^, branched-chain amino acids^11^, and lactate^12,13^ can be the fuels for cancer. Among these nutrients, the fatty acid is the preferred one for energy storage as it can generate twice as much energy as carbohydrates from the same dry mass^17^. Here, our data validate the crucial role of long-chain fatty acids in stress response in cancer cells under GS (Fig. 5). Indeed, our findings are generally consistent with other studies showing that fatty acid oxidation promotes tumor growth under metabolic stress^8,18,50,62^. Moreover, the consumption of fatty acids, obtained from the extracellular media or hydrolyzed from triglycerides in lipid droplets, are controlled by the transportation process into mitochondria via the carnitine palmitoyltransferase (CPT) system^17^. In this study, we confirm the crucial role of ACC2 in regulating long-chain fatty acid oxidation via CPT1 and discover the PFKP/AMPK/ACC2/CPT1 signaling pathway which links PFKP, a key glycolysis enzyme, to fatty acid oxidation under metabolic stress.

In this study, several questions remain to be further investigated. First, since AMPK is a protein kinase, PFKP may be a substrate of AMPK, as indicated by their enhanced interaction under GS (Fig. 2). Although our data suggest that the PFKP-AMPK interaction is independent of AMPK kinase activity (Fig. 3a, b) and no strong evidence of phosphorylation site on PFKP is found, more efforts are still needed to examine this possibility. Second, the detailed mechanisms underlying the enhanced PFKP-AMPK interaction under GS are not clear. Current data demonstrate that the interaction is triggered by the reduced level of F6P or FBP upon GS (Fig. 3c, d), but more convincing evidence such as finding a mutation in PFKP that interfered with binding to AMPK is desirable. Besides, the levels of glycolysis metabolites decrease very rapidly upon GS^25^, but we observe that the PFKP-AMPK interaction is not enhanced until two-hour GS (Fig. 2b), indicating that other unknown factors may affect the interaction. What we have found so far is that the interaction can be rapidly disrupted by F6P or FBP in an in vitro experiment (Fig. 3d), but more efforts are required to address the gap of delayed PFKP-AMPK interaction upon GS. Meanwhile, although the regulatory domain of PFKP is identified to mediate its interaction with AMPK (Fig. 2i), the interacting region of AMPK remains to be further examined. Lastly, whether the PFKP-AMPK signaling pathway could serve as a potential therapeutic target in cancer, especially in NSCLC, remains to be further investigated. The development of PFKP inhibitors may suppress the oncogenic function of PFKP in cancer, but more experiments are needed to validate this hypothesis.

In summary, we uncover a novel mechanism through which PFKP alleviates GS-induced metabolic stress in NSCLC cells. As illustrated in Fig. 7, PFKP mediates the mitochondrial recruitment of AMPK and results in enhanced ACC2 phosphorylation, which facilitates long-chain fatty acid oxidation to maintain energy and redox homeostasis and promote cancer cell survival under GS. This non-glycolysis-related function of PFKP provides new opportunities in developing novel therapeutics in cancer.

## Material and methods

### Reagents

Glucose (G8270), Glucose-6-Phosphate (G7250), Fructose-6-Phosphate (F3627), Fructose-1,6-bisphosphate (F6803), Phosphoenolpyruvic acid (860077), Pyruvate (S8636), Lactate (PHR1113), Propidium iodide (P4170), and N-acetylcysteine (A9165) were purchased from Sigma-Aldrich. Dihydroxyacetone-Phosphate (sc-214896), Glyceraldehyde-3-Phosphate (sc-280680), 2/3-Phosphoglyceric acid (sc-397368), AICAR (sc-200659), and paraformaldehyde (sc-281692) were purchased from Santa Cruz. Puromycin dihydrochloride (A1113803) and Hygromycin B (10687010) were purchased from Thermo Fisher. 2,5-Anhydro-D-glucitol-1,6-diphosphate (TRC-A648020) was purchased from Scientific Resources Pte Ltd.

### Plasmids, siRNA, and transfection

The CRISPR-Cas9 system pSpCas9(BB)-2A-Puro (PX459) V2.0 (Addgene, 62988) was used to knock out PFKP. The sgRNA oligonucleotides targeting human PFKP gene were 5’- CACCGGATGTGTGTCAAACTCTCGG (forward) and 5’- AAACCCGAGAGTTTGACACACATCC (reverse). The vector pcDNA3.1-FLAG-HA-Hygro(-) was used for re-constitution of PFKP and domains in PFKP KO cells. The cDNA of human PFKP was cloned from HEK293 cells with primers: 5’-CTCTAGAATGGACGCGGACGACT (forward) and 5’-TTAAGCTTTCAGACACTCCAGGGCTG (reverse). Then, the nucleotides 5’-CT**C TCG** GA **G** (Leu-Ser-Glu) targeted by CRISPR-Cas9 system was replaced by 5’-CT**A AGT** GA**A** (Leu-Ser-Glu) with primers: 5’-GAGGAGCAGATGTGTGTCAAACTAAGTGAAAACCGTGCCCGGAAAAAAAGGCTG (forward) and 5’-CAGCCTTTTTTTCCGGGCACGGTTTTCACTTAGTTTGACACACATCTGCTCCTC (reverse). Primers for domain 1 were 5’-ACTTAGGATCCATGGACGCGGACGACTCC (forward) and 5’-GGTATCTCGAGTCAGATGGCAAGTCGCTTGTAGGTGT (reverse). Primers for domain 2 were 5’-ACTTAGGATCCATGAACGTAGCTGTCATCAACGTGGG (forward) and 5’-GGTATCTCGAGTCAGACACTCCAGGGCTGC (reverse). The plasmid pLV-mitoGFP (Addgene, #44385) was a gift from Dr. Zhai’s lab^63^. The PFKP siRNA (sc-106401), ACC2 siRNA (sc-43597), siVDAC1 (sc-42355), siVDAC2 (sc-42357), siAMPKα1/2 (sc-45312), and control siRNA (sc-37007) were purchased from Santa Cruz. The lipofectamine 3000 (Thermo Fisher, L3000015) and lipofectamine RNAiMAX (Thermo Fisher, 13778150) were used for plasmids and siRNA transfections, respectively.

### Cell lines and cell culture

The human lung cancer cell lines of H1299, H1703, and H358 were obtained from American Type Culture Collection (ATCC). The human PFKP gene was knocked out by using the CRISPR-Cas9 system. Cells transfected with the empty vector or the vector with sgRNA oligonucleotides were selected with 1 µg/mL puromycin. The PFKP KO clones were verified by immunoblotting. In the PFKP KO cells, the human PFKP was reconstituted by using the vector pcDNA3.1-FLAG-HA-Hygro(-). Cells transfected with the empty vector or the vector with designed PFKP cDNA were selected with 300 µg/mL hygromycin. The expression of PFKP was verified by immunoblotting. All cells were cultured in Dulbecco’s High Glucose Modified Eagles Medium (high-glucose DMEM, HyClone, SH3002201) containing 10% fetal bovine serum (HyClone, SV30160.03) in a 5% CO_2_ atmosphere at 37 °C. In glucose starvation experiments, cells were cultured in the glucose-free medium which was composed of no-glucose DMEM (without glucose or sodium pyruvate but with 4 mM glutamine, Gibco, #11966025) with 10% dialyzed fetal bovine serum (Gibco, #26400044). In the control group, 25 mM glucose was added into the glucose-free medium.

### Western blotting

Cells were washed twice with cold PBS, lysed in the SDS lysis buffer (62.5 mM Tris, pH 6.8, 2% SDS, 1 mM EDTA, 20% glycerol, 2 mM dithiothreitol, and 1× Halt™ Protease and Phosphatase Inhibitor Cocktail), and boiled for 10 min. Protein concentration was determined by the DC protein assay method (Bio-Rad). The protein sample was mixed with 2× or 4× Laemmli sample buffer (BIO-RAD, #1610737, #1610747), resolved by SDS-PAGE, and transferred onto PVDF membrane (Bio-Rad, #1620177). Then, membranes were blocked with 5% non-fat milk (Bio-Rad Laboratories, 1706404) for one hour, incubated with indicated primary antibodies overnight at 4 °C, and incubated with HRP-conjugated secondary antibodies for two h at room temperature. Finally, the chemiluminescence signals were detected by ImageQuant LAS500 (GE Healthcare) with the enhanced chemiluminescence reagent (GE Healthcare, RPN2235). The antibodies ACC (#3676), ACC1 (#4190), ACC2 (#8578), p-ACC (Ser79, #11818), AMPKα (#5831), p-AMPKα (Thr172, #2535), AMPKβ1/2 (#4150), Calreticulin (#12238), Anti-rabbit IgG HRP-linked (#7074), and Anti-mouse IgG HRP-linked (#7076) were purchased from Cell Signaling Technology. PFKP antibody (ab204131) was purchased from Abcam. VDAC antibody (10866-1-AP), His-tag antibody (66005-1-Ig) and GAPDH antibody (60004-1-Ig) were purchased from Proteintech. LAMP1 antibody (sc-19992), PFKM (sc-166722), and PFKL (sc-393713) were purchased from Santa Cruz. α-Tubulin antibody (T6199) and Flag antibody (A8592) were purchased from Sigma-Aldrich. Pan-myristoylation (STJ98666) antibody was purchase from St John’s laboratory. All the primary antibodies were diluted according to the protocols in tris buffered saline with 0.1% (v/v) Tween 20 (TBST) with 5% (w/v) bovine serum albumin and 0.1% (w/v) sodium azide. The secondary HRP-linked antibodies were 1:3000 diluted in TBST with 5% (w/v) non-fat milk.

### Immunoprecipitation (IP)

Cells in a 15 cm dish (80% confluence) were washed twice with cold PBS and lysed in 1 mL Pierce™ IP Lysis Buffer (Thermo Fisher, #87787, 25 mM Tris hydrochloric acid, pH 7.4, 150 mM NaCl, 1% NP-40, 1 mM EDTA, 5% glycerol) with 1× Halt™ Protease and Phosphatase Inhibitor Cocktail on ice for 30 min with periodic mixing. The lysates were then transferred to a tube and centrifuged at 17,000× *g* for 15 min at 4 °C followed by the protein concentration determination. 100 ng proteins from the supernatants were aliquoted as the input. The remaining supernatants were precleared with 10 μL mouse IgG (sc-2343) overnight at 4 °C and centrifuged at 17,000× *g* for 15 min at 4 °C. The supernatants were further mixed with 10 μL agarose conjugated antibodies or mouse IgG for 3 h at 4 °C. Finally, the beads were washed thrice with 1 mL IP lysis buffer, boiled with 50 μL 2× Laemmli sample buffer for 10 min, and retained for western blotting analysis. The following agarose conjugated antibodies for IP were purchased from Santa Cruz: AMPKα (sc-74461 AC), PFKP (sc-514824 AC), ACC1 (sc-137104 AC), ACC2 (sc-390344 AC), and mouse IgG (sc-2343). The FLAG M2 monoclonal antibody affinity gel (A2220) for IP of Flag-tagged proteins was purchase from Sigma-Aldrich.

### Protein-protein interaction assay

Flag-HA tagged PFKP, domain 1, and domain 2 were purified by Flag IP. H1299 cells transfected with empty vector, Flag-HA tagged PFKP, domain 1, or domain 2 in a 60-mm dish were washed twice with cold PBS and lysed in 1 mL lysis buffer (20 mM Tris hydrochloric acid, pH 7.4, 150 mM NaCl, 1 mM EDTA, 1 mM EGTA, 1% Triton X-100) with 1× Halt™ Protease and Phosphatase Inhibitor Cocktail on ice. The lysates were sonicated with a vibrating probe on ice and centrifuged at 17,000× *g* for 15 min at 4 °C. The supernatants were pre-cleared with 10 μL mouse IgG (sc-2343) overnight at 4 °C and centrifuged at 17,000× *g* for 15 min at 4 °C. The supernatants were further mixed with 10 μL FLAG M2 monoclonal antibody affinity gel (Sigma-Aldrich, A2220) for 3 h at 4 °C. The beads were then washed thrice with 1 mL lysis buffer. The purified protein on the beads was incubated with 50 ng His tagged AMPKα1/β1/γ2 (Thermo Fisher, #PV6238) or GST-His tagged AMPKα2/β2/γ3 (Thermo Fisher, #A30488) in 1 mL lysis buffer for 1 h at room temperature. After incubation, the beads were washed thrice with 1 mL lysis buffer and subjected to western blotting analysis.

### Mass spectrometry-based proteomics analysis

H460 cells treated with GS for 4 h were subjected to AMPKα IP. The immunoprecipitated proteins on the agarose beads were digested for mass spectrometry analysis. H1299 cells transfected with Flag-HA tagged PFKP were treated with GS for 4 h and subjected to Flag IP. The immunoprecipitated proteins were boiled with 20 μL 2× Laemmli sample buffer and loaded into an SDS-PAGE gel. Proteins in the gel were extracted and digested for mass spectrometry analysis as previously described^64^.

### Proximity ligation assay (PLA) and confocal microscopy

Cells cultured on the coverslip were washed twice with PBS, fixed with 4% paraformaldehyde in PBS for 15 min, and permeabilized with 0.25% Triton X-100 in PBS for 15 min. PLA was performed on the permeabilized cells by using the Duolink™ In Situ Red Starter Kit (Sigma-Aldrich, DUO92101) following the manufacturer’s instruction. Cells were blocked with the blocking buffer for 1 h at 37 °C, incubated with rabbit anti-AMPKβ1/2 (Cell Signaling Technology, #4150) and mouse anti-PFKP antibodies (Santa Cruz, sc-514824) overnight at 4 °C, and treated with a pair of oligonucleotide-labeled probes for 30 min at 37 °C. If the distance between two proteins was less than 40 nm, connector oligos joined the probes and ligated upon the incubation with ligase enzyme for 60 min at 37 °C. The ligated circle oligonucleotide template was further amplified through polymerase-catalyzed rolling-circle reactions for 100 min at 37 °C. After the amplification, the coverslips were washed with buffers in the kit and mounted with the ProLong™ Diamond Antifade Mountant with DAPI (Thermo Fisher, P36962). Last, the cells were visualized under confocal microscope (Olympus Fluoview FV1000, Olympus America Inc., PA). The original data were analyzed by ImageJ (NIH). For the quantification of puncta in PLA, fluorescence signals from five layers through *z*-direction scanning were compressed into one image. The puncta in the images were counted by the “Analyze Particle” plugin. For the co-localization analysis of PLA puncta and mitochondria, the fluorescence signals of PLA and mito-GFP in one layer were captured simultaneously. Then, the gray values of each layer in interested areas were recorded by “Plot Profile” function to evaluate the co-localization of mito-GFP and PLA puncta.

### Immunofluorescence staining and confocal microscopy

Cells cultured on the coverslip were washed twice with PBS, fixed with 4% paraformaldehyde in PBS for 15 min, and permeabilized with 0.25% Triton X-100 in PBS for 15 min. The permeabilized cells on the coverslip were incubated with respective primary antibody overnight at 4 °C. Then, the cells were washed thrice with PBS and incubated with Alexa Fluor secondary antibody (1:300, Invitrogen) for 2 h at room temperature. The cells were washed twice with PBS and mounted with the ProLong™ Diamond Antifade Mountant with DAPI (Thermo Fisher, P36962). Last, the cells were visualized under confocal microscope (Olympus Fluoview FV1000, Olympus America Inc., PA) and the original data were processed by ImageJ (NIH).

### Mitochondria and cytosol fractionation

A two-step strategy was adopted to isolate mitochondria in cells while the cytosol fraction was purified simultaneously. The first step was sucrose density gradient centrifugation as previously described^65^. Briefly, cells from three 15-cm dishes (80% confluence) were collected and homogenized by a Dounce homogenizer (Wheaton, #357538) for 150 strokes on ice in 1 mL separation buffer (250 mM sucrose, 5 mM MgCl_2_, 50 mM Tris hydrochloric acid 7.4, and 1× Halt™ Protease and Phosphatase Inhibitor Cocktail). The samples were then centrifuged twice at 800× *g* for 10 min at 4 °C to yield crude supernatants of mitochondria. The supernatants were further centrifuged at 11,000× *g* for 15 min at 4 °C to yield crude pellets of mitochondria. The supernatants were further centrifuged twice at 20,000× *g* for 20 min at 4 °C to yield the purified cytosol fraction. Meanwhile, the pellets containing crude fraction of mitochondria were resuspended twice in 1.5 mL separation buffer and centrifuged at 11,000× *g* for 15 min at 4 °C. The crude mitochondria pellets were then subjected to the second step of mitochondria fractionation by using the Mitochondria Isolation QuadroMACS Kit (Miltenyi Biotec). Briefly, the mitochondria pellets were resuspended in 1 mL lysis buffer and 9 mL separation buffer from the kit followed by rotating with Anti-TOM22 magnetic beads in 4 °C for 2 h. The samples were then loaded on a column in a magnetic field to retain the magnetically labeled mitochondria, followed by twice washing with 3 mL separation buffer. After washing, the mitochondria in the column were flushed out with 1.5 mL separation buffer without the magnetic field. The beads-conjugated mitochondria were centrifuged at 13,000× *g* for 15 min at 4 °C, followed by thrice washing with 1.5 mL storage buffer. Finally, the centrifuged mitochondria were lysed in the SDS lysis buffer for western blotting analysis.

### Measurement of fatty acids by mass spectrometry

Six saturated fatty acids (free and esterified forms in total) were measured by gas chromatography-tandem mass spectrometry (GC-MS/MS) as previously described^66^ with some modifications. Briefly, trypsinized cells from a 10-cm dish (80% confluence) were collected, washed twice with cold PBS, and centrifuged at 600× *g* for 3 min at 4 °C. Cells were resuspended in 1.5 mL (control group) or 1 mL (GS group) cold PBS. 50 μL of the cells were lysed in SDS lysis buffer to determine the protein concentration, while 950 μL of cells were centrifuged and stored in −80 °C. The remaining cells in each sample were mixed and aliquoted as quality control samples. For GC-MS/MS analysis, the cell pellet was homogenized by TissueLyser LT (Qiagen) at 25 Hz for 10 min in 1 mL NaOH-methanol solution (0.5 M) with 1 mg/L stable-isotope-labeled fatty acid mixtures (CLM-8455, Cambridge Isotope Laboratories) as the internal standards. The mixture was further sonicated for 10 min and heated at 80 °C for 10 min to hydrolyze esterified fatty acids. After centrifugation at 14,000 rpm for 20 min at 4 °C, 500 μL supernatant was transferred into a glass vial for methylation by BF3-MeOH. After methylation, 0.5 mL hexane and 0.3 mL saturated NaCl solution were added, and the mixture was vortexed for 3 min and centrifuged at 3000 rpm for 3 min. Then, hexane layer was analyzed by an Agilent 7890 GC system coupled to a 7000B QQQ mass detector equipped with a chemical ionization source. Samples were analyzed in a random order with quality control samples inserted randomly throughout each analytical batch. Finally, the fatty acid concentration was calibrated by protein concentration for each sample.

### Bioinformatics analysis of LUAD datasets

The NIH LUAD dataset with processed microarray data^41^ were used to compare the expression of PFKP in well, moderate, and poor differentiated tumors. The TCGA LUAD dataset were downloaded from c-Bioportal (https://www.cbioportal.org/datasets, LUAD, “PanCancer Atlas”). In this dataset, the RNA-seq data were processed and normalized accordingly^67^ to analyze the expression levels of PFKP, PFKL, and PFKM in tumor and adjacent normal tissue. To estimate whether the expression of genes of interest was significantly associated with cancer patient’s survival, we adopted the one-dimensional data-driven grouping method^68^ for the survival analysis. Briefly, (i) the clinical data were sorted by the gene expression values; (ii) the values were fitted to survival times and events using the Cox proportional hazards model; (iii) goodness-of-fit analysis was applied to get the separation between the sorted patients into low- and high-risk subgroups; (iv) Cox hazards model and Wald test statistic were used to calculate the differences between the Kaplan-Meier survival curves.

### Measurement of long-chain free fatty acids, ATP, and NADPH

The concentration of long-chain free fatty acids (C8 and longer), ATP, NADPH were determined by the Free Fatty Acid Quantitation Kit (Sigma-Aldrich, MAK044), ATP Colorimetric/Fluorometric Assay Kit (BioVision, #K354), and NADP/NADPH Quantification Colorimetric Kit (BioVision, #K347) following the manufacturer’s instruction, respectively.

### Measurement of ROS

The intracellular ROS level was determined by the CellROX Green Reagent (ThermoFisher, C10444) following the manufacturer’s instruction. The medium fluorescence intensity (MFI) of ten thousand cells was used to evaluate the ROS level.

### Evaluation of cell viability

Propidium iodide (PI) exclusion test coupled with flow cytometry was applied to evaluate cell viability as previously described^7^. The data were processed by the R package ‘flowCore’ to calculate the proportion of live cells.

### Statistical analysis

All image and western blotting data were representative from at least three independent experiments. All numeric data were presented as means ± SD and analyzed by the Student’s *t*-test or Mann–Whitney U test according to the type and characteristics of the data using R programming. The *P* value less than 0.05 (*), 0.01 (**), or 0.001 (***) was considered as statistically significant.

## Supplementary information

Supplementary Information

## Data Availability

All data needed to support the conclusions in the paper are present in the paper and/or the Supplementary Materials. Additional data related to this paper may be requested from the authors.
