# Supplementary material for: PFKP alleviates glucose starvation-induced metabolic stress in lung cancer cells via AMPK-ACC2 dependent fatty acid oxidation
Source: Cell Discov. 2022 May 31;8:52. doi: 10.1038/s41421-022-00406-1 (PMC9156709; doi:10.1038/s41421-022-00406-1)
Supplement: Supplementary file 1 — Supplementary Information [file 41421_2022_406_MOESM1_ESM.pdf]

Supplementary information

PFKP alleviates glucose starvation-induced metabolic stress in lung cancer cells via AMPK-ACC2 dependent fatty acid oxidation

Jiaqing Chen, Li Zou, Guang Lu, Oleg Grinchuk, Lei Fang, Derrick Sek Tong Ong, Reshma Taneja, Choon-Nam Ong, and Han-Ming Shen*

This file includes supplementary Fig. S1-S7, and Table S1-S2.

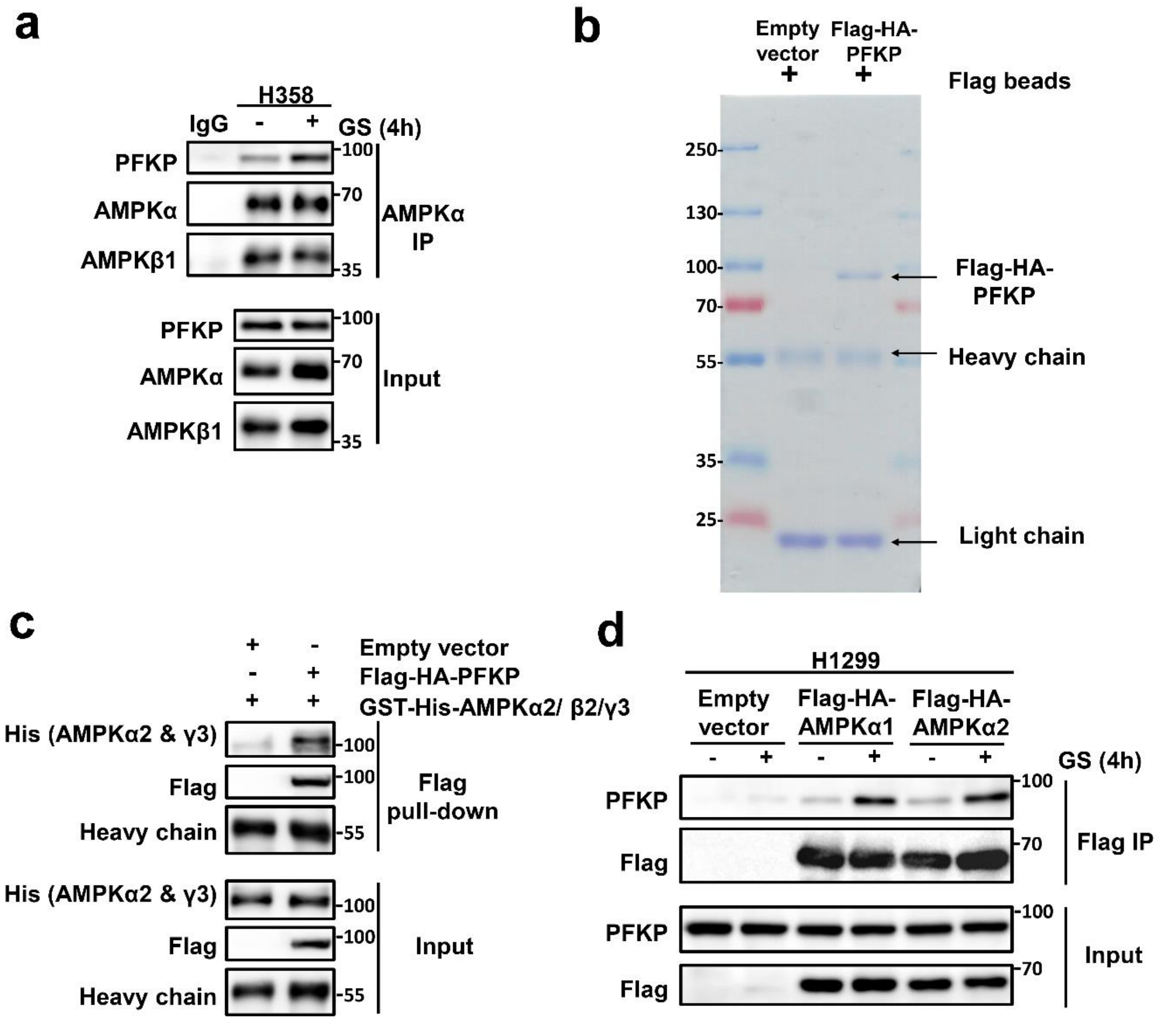

Fig. S1 GS enhances the direct interaction between PFKP and AMPK. a Immunoprecipitation (IP) of AMPKα in H358 under control or GS. **b** Coomassie blue staining of purified PFKP on Flag beads. **c** *in vitro* protein-protein interaction assay of purified PFKP and AMPK. Flag-HA tagged PFKP was purified by Flag IP and then incubated with GST-His-tagged AMPKα2/β2/γ3 for 1 h at room temperature. **d** Flag IP in H1299 cells transfected with empty vector, Flag-HA tagged AMPKα1, or Flag-HA tagged AMPKα2 under control or GS.

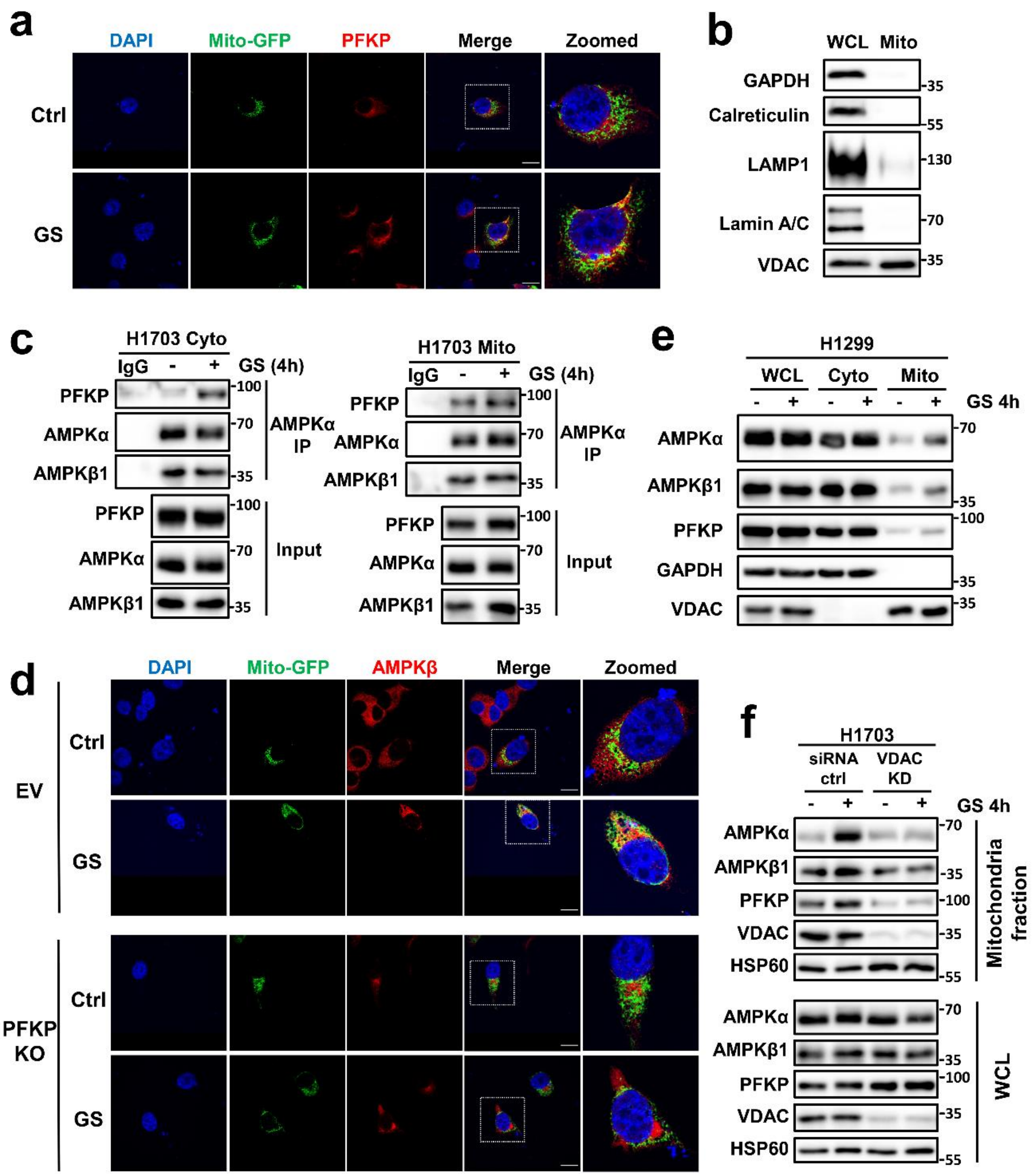

Fig. S2 PFKP promotes mitochondrial recruitment of AMPK. a Immunofluorescence analysis of mito-GFP and PFKP in H1299 cells under control (Ctrl) or GS for 4 h. Scale bar: 10 µm. **b** Mitochondria fractionation (Mito) of H1299 cells by sucrose density gradient centrifugation and Mitochondria Isolation QuadroMACS Kit. GAPDH: a cytosol marker; Calreticulin: an ER marker; LAMP1: a lysosome marker; Lamin A/C: a nuclear marker; VDAC: a mitochondria marker. **c** AMPKα IP in cytosol fraction (Cyto) and mitochondria fractionation (Mito) of H1703 cells respectively under control or GS. **d** Immunofluorescence analysis of mito-GFP and AMPK in H1299 cells and H1299 PFKP KO cells under control (Ctrl) or GS for 4 h. Scale bar: 10 µm. **e** Analysis of protein levels in cytosol fraction (Cyto) and mitochondria fractionation (Mito) of H1703 cells under control or GS. **f** Mitochondria fraction analysis of H1703 cells with or without VDAC knock-down under control or GS.

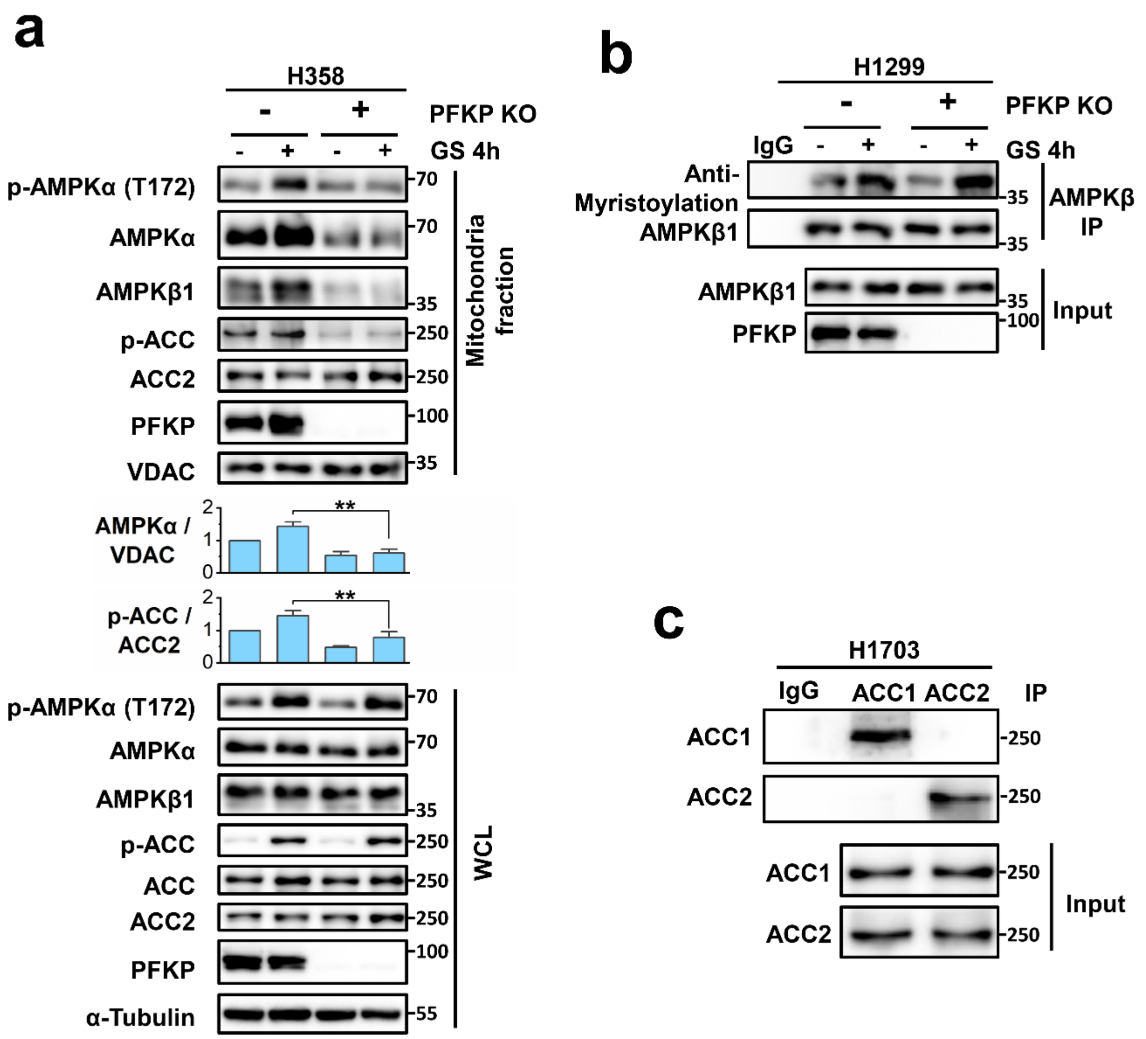

Fig. S3 PFKP promotes mitochondrial recruitment of AMPK and enhances the phosphorylation of ACC2. a. Mitochondria fraction analysis of H358 cells under control or GS. Data are shown as mean ± SD with three biological replicates. **p* < 0.05, ***p* < 0.01, ****p* < 0.001 by two-tailed Student's t-test. **b** Myristolylation of AMPKβ1 under control or GS using AMPKβ1 IP and myristoylation antibody in H1299 cells. **c** Validation of the ACC1 and ACC2 IP in H1703 cells.

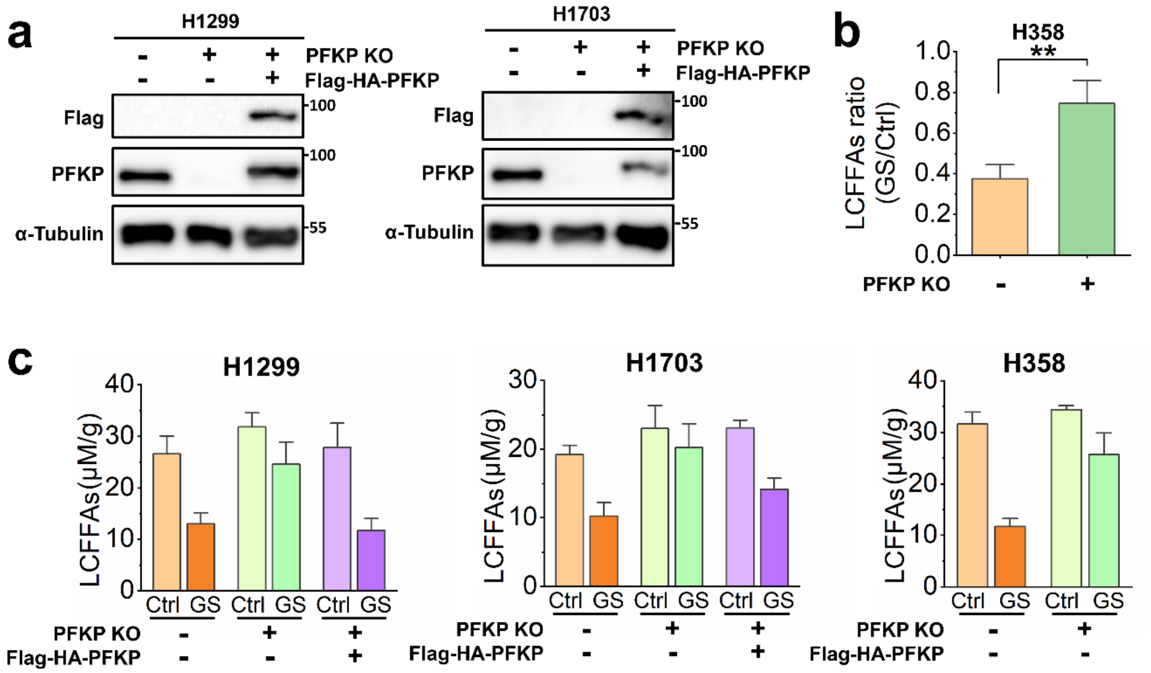

Fig. S4 PFKP promotes long-chain fatty acid oxidation via ACC2. a PFKP KO and reconstitution of Flag-HA tagged PFKP in H1299 and H1703 cell lines. **b** Relative level of long chain free fatty acids (LCFFAs) in H358 cells (n = 4) under GS for 14 h versus control. Data are shown as mean ± SD with n indicating the number of biological replicates. *$p < 0.05$, **$p < 0.01$, ***$p < 0.001$ by two-tailed Student's t-test. **c** Absolute level of long chain free fatty acids (LCFFAs) in H1299, H1703, and H358 cells (n = 4) under control or GS for 14 h. Data are shown as mean ± SD with n indicating the number of biological replicates.

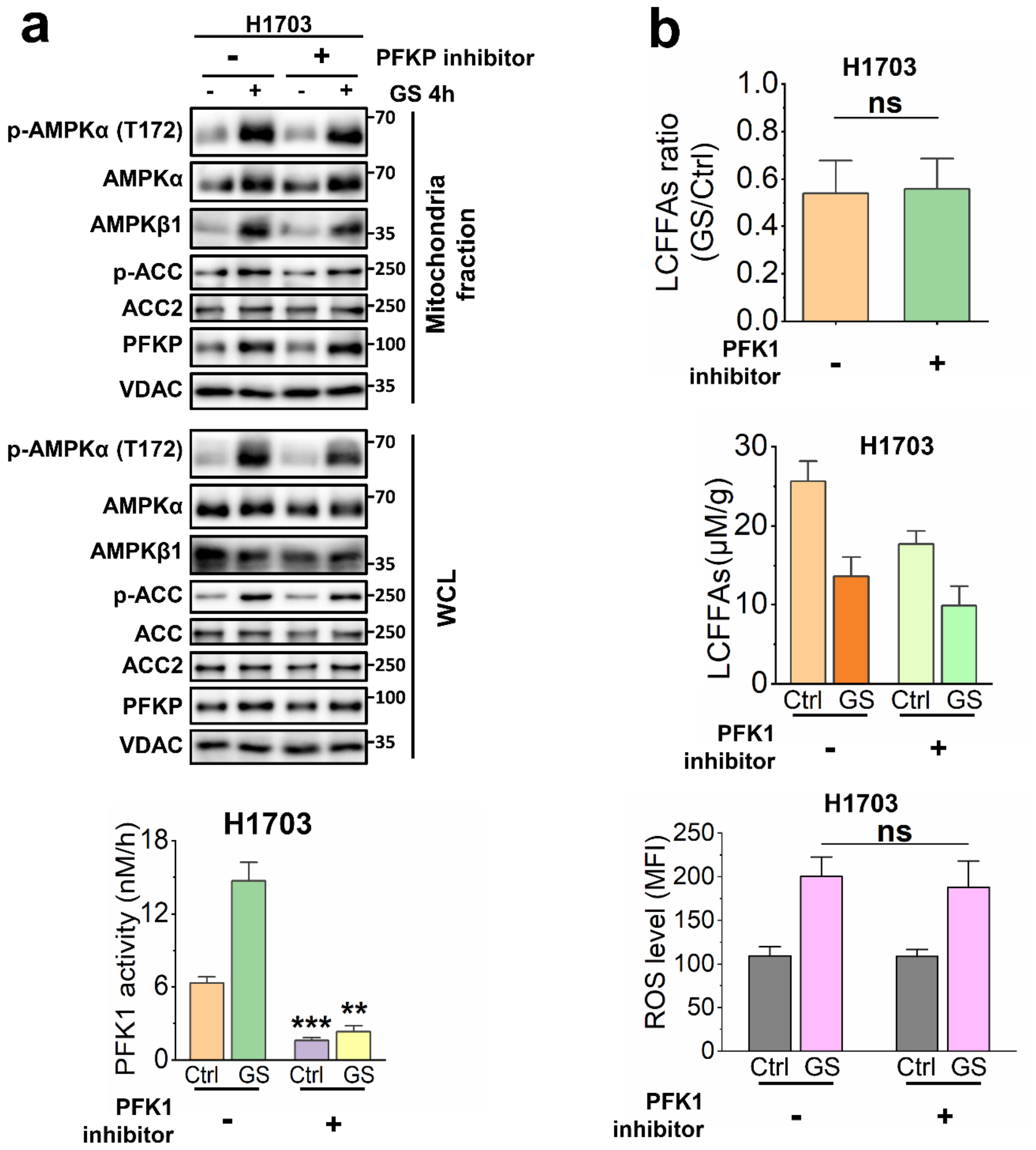

Fig. S5 The regulatory effects of PFKP are independent of its enzyme activity. a Mitochondria fraction analysis of H1703 cells treated with or without 1mM PFKP inhibitor 2,5-Anhydro-D-glucitol-1,6-diphosphate under control or GS. The enzyme activity was measured by the Phosphofructokinase Activity Colorimetric Assay Kit (K776, BioVision). **b** Relative and absolute level of long chain free fatty acids (LCFFAs) and relative level of ROS in H1703 cells ($n = 4$) treated with or without 1mM PFKP inhibitor 2,5-Anhydro-D-glucitol-1,6-diphosphate under control or GS for 14 h. Data are shown as mean ± SD with n indicating the number of biological replicates. ns: no significance, *$p < 0.05$, **$p < 0.01$, ***$p < 0.001$ by two-tailed Student's t-test.

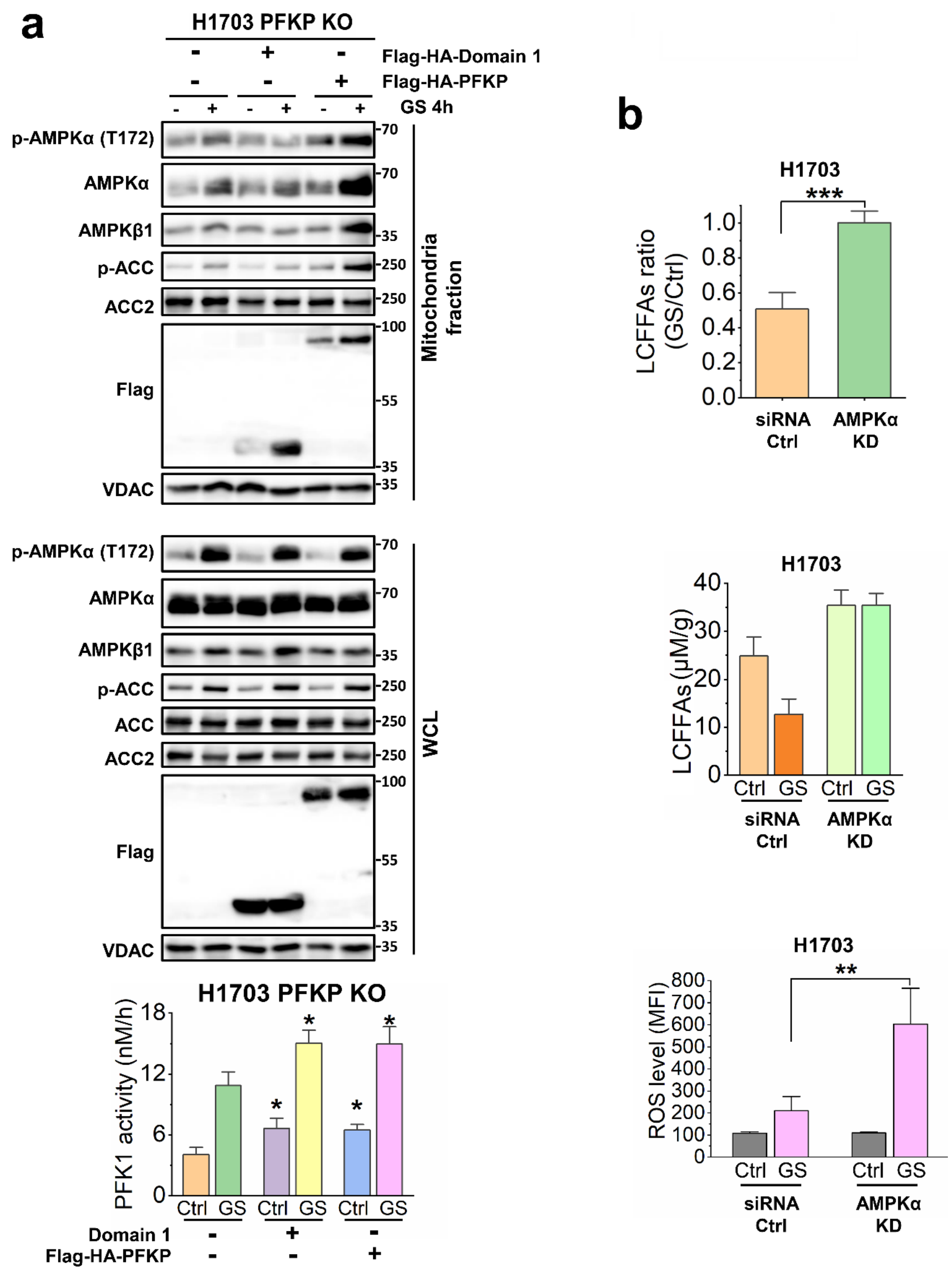

Fig. S6 The regulatory effects of PFKP are independent of the catalytic domain, but dependent on AMPK. a Mitochondria fraction analysis of H1703 PFKP KO cells transfected with empty vector, Domain 1, or Flag-HA tagged PFKP under control or GS.

The enzyme activity was measured by the Phosphofructokinase Activity Colorimetric Assay Kit (K776, BioVision). **b** Relative and absolute level of long chain free fatty acids (LCFFAs) and relative level of ROS in H1703 cells ($n = 4$) with AMPKα KD under control or GS for 14 h. Data are shown as mean ± SD with n indicating the number of biological replicates. *$p < 0.05$, **$p < 0.01$, ***$p < 0.001$ by two-tailed Student's t-test.

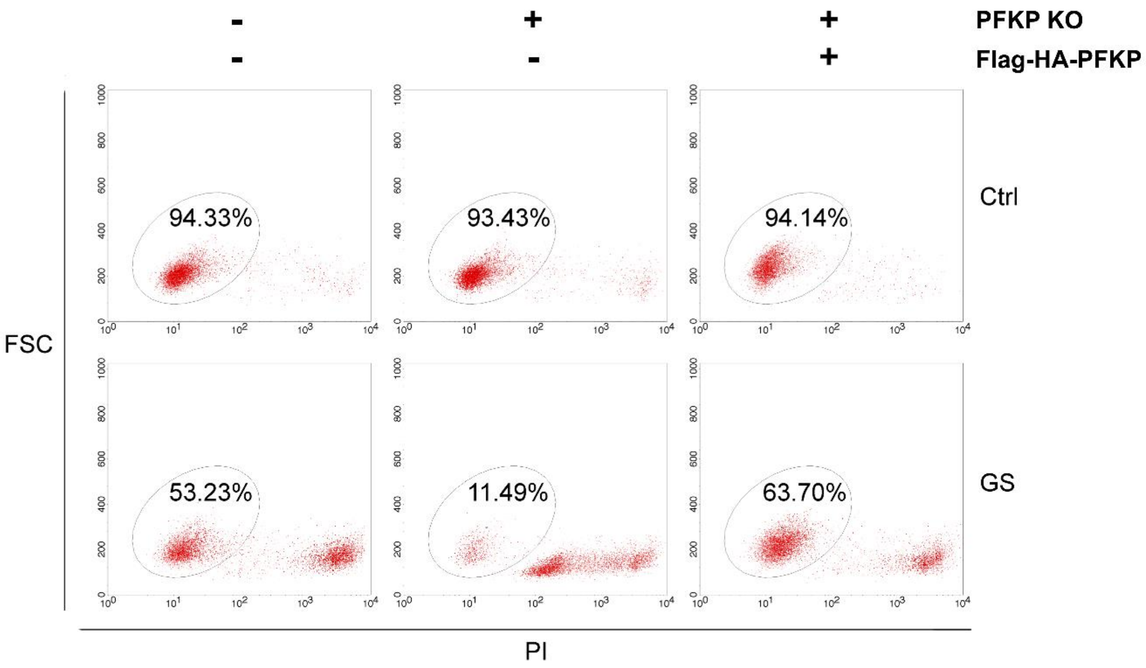

Fig. S7 PFKP promotes NSCLC cell survival under GS. Propidium iodide (PI) exclusion test of H1703 cells under control (Ctrl) or GS for 48 h. FSC: forward scatter. The number shown in each panel represents the percentage of viable cells with less PI uptake.

Table S1. Mass spectrometry analysis of interacting proteins using AMPKα as bait

Accession	Description	#Peptides	
Q13131	5'-AMP-activated protein kinase catalytic subunit alpha-1 OS=Homo sapiens GN=PRKAA1 PE=1 SV=4 - [AAPK1_HUMAN]	7	**AMPK subunites**
O43741	5'-AMP-activated protein kinase subunit beta-2 OS=Homo sapiens GN=PRKAB2 PE=1 SV=1 - [AAKB2_HUMAN]	3	
P54619	5'-AMP-activated protein kinase subunit gamma-1 OS=Homo sapiens GN=PRKAG1 PE=1 SV=1 - [AAKG1_HUMAN]	3	
Q01813	ATP-dependent 6-phosphofructokinase, platelet type OS=Homo sapiens GN=PFKP PE=1 SV=2 - [PFKAP_HUMAN]	4	**Glycolytic enzymes and known targets**
P52789	Hexokinase-2 OS=Homo sapiens GN=HK2 PE=1 SV=2 - [HXK2_HUMAN]	4	
P14618	Pyruvate kinase PKM OS=Homo sapiens GN=PKM PE=1 SV=4 - [KPYM_HUMAN]	13	
O43175	D-3-phosphoglycerate dehydrogenase OS=Homo sapiens GN=PHGDH PE=1 SV=4 - [SERA_HUMAN]	9	
Q06210	Glutamine--fructose-6-phosphate aminotransferase [isomerizing] 1 OS=Homo sapiens GN=GFPT1 PE=1 SV=3 - [GFPT1_HUMAN]	6	
P04406	Glyceraldehyde-3-phosphate dehydrogenase OS=Homo sapiens GN=GAPDH PE=1 SV=3 - [G3P_HUMAN]	6	
P00338	L-lactate dehydrogenase A chain OS=Homo sapiens GN=LDHA PE=1 SV=2 - [LDHA_HUMAN]	4	
Q13085	Acetyl-CoA carboxylase 1 OS=Homo sapiens GN=ACACA PE=1 SV=2 - [ACACA_HUMAN]	3	
P09211	Glutathione S-transferase P OS=Homo sapiens GN=GSTP1 PE=1 SV=2 - [GSTP1_HUMAN]	4	
P60709	Actin, cytoplasmic 1 OS=Homo sapiens GN=ACTB PE=1 SV=1 - [ACTB_HUMAN]	8	
P23526	Adenosylhomocysteinase OS=Homo sapiens GN=AHCY PE=1 SV=4 - [SAHH_HUMAN]	3	
P05141	ADP/ATP translocase 2 OS=Homo sapiens GN=SLC25A5 PE=1 SV=7 - [ADT2_HUMAN]	7	
P07355	Annexin A2 OS=Homo sapiens GN=ANXA2 PE=1 SV=2 - [ANXA2_HUMAN]	8	
Q99700	Ataxin-2 OS=Homo sapiens GN=ATXN2 PE=1 SV=2 - [ATX2_HUMAN]	10	
P25705	ATP synthase subunit alpha, mitochondrial OS=Homo sapiens GN=ATP5A1 PE=1 SV=1 - [ATPA_HUMAN]	3	
O14497	AT-rich interactive domain-containing protein 1A OS=Homo sapiens GN=ARID1A PE=1 SV=3 - [ARI1A_HUMAN]	19	

Q8NFD5	AT-rich interactive domain-containing protein 1B OS=Homo sapiens GN=ARID1B PE=1 SV=2 - [ARI1B_HUMAN]	14	
O95429	BAG family molecular chaperone regulator 4 OS=Homo sapiens GN=BAG4 PE=1 SV=1 - [BAG4_HUMAN]	5	
P07814	Bifunctional glutamate/proline--tRNA ligase OS=Homo sapiens GN=EPRS PE=1 SV=5 - [SYEP_HUMAN]	10	
P27708	CAD protein OS=Homo sapiens GN=CAD PE=1 SV=3 - [PYR1_HUMAN]	39	
Q14444	Caprin-1 OS=Homo sapiens GN=CAPRIN1 PE=1 SV=2 - [CAPR1_HUMAN]	9	
P31327	Carbamoyl-phosphate synthase [ammonia], mitochondrial OS=Homo sapiens GN=CPS1 PE=1 SV=2 - [CPSM_HUMAN]	10	
O00299	Chloride intracellular channel protein 1 OS=Homo sapiens GN=CLIC1 PE=1 SV=4 - [CLIC1_HUMAN]	7	
Q00610	Clathrin heavy chain 1 OS=Homo sapiens GN=CLTC PE=1 SV=5 - [CLH1_HUMAN]	32	
Q14677	Clathrin interactor 1 OS=Homo sapiens GN=CLINT1 PE=1 SV=1 - [EPN4_HUMAN]	3	
Q10570	Cleavage and polyadenylation specificity factor subunit 1 OS=Homo sapiens GN=CPSF1 PE=1 SV=2 - [CPSF1_HUMAN]	7	
Q9P2I0	Cleavage and polyadenylation specificity factor subunit 2 OS=Homo sapiens GN=CPSF2 PE=1 SV=2 - [CPSF2_HUMAN]	6	
Q9UKF6	Cleavage and polyadenylation specificity factor subunit 3 OS=Homo sapiens GN=CPSF3 PE=1 SV=1 - [CPSF3_HUMAN]	4	
O43809	Cleavage and polyadenylation specificity factor subunit 5 OS=Homo sapiens GN=NUDT21 PE=1 SV=1 - [CPSF5_HUMAN]	4	
Q16630	Cleavage and polyadenylation specificity factor subunit 6 OS=Homo sapiens GN=CPSF6 PE=1 SV=2 - [CPSF6_HUMAN]	6	
Q8N684	Cleavage and polyadenylation specificity factor subunit 7 OS=Homo sapiens GN=CPSF7 PE=1 SV=1 - [CPSF7_HUMAN]	3	
Q05048	Cleavage stimulation factor subunit 1 OS=Homo sapiens GN=CSTF1 PE=1 SV=1 - [CSTF1_HUMAN]	4	
Q12996	Cleavage stimulation factor subunit 3 OS=Homo sapiens GN=CSTF3 PE=1 SV=1 - [CSTF3_HUMAN]	3	
P21291	Cysteine and glycine-rich protein 1 OS=Homo sapiens GN=CSRP1 PE=1 SV=3 - [CSRP1_HUMAN]	3	

P22695	Cytochrome b-c1 complex subunit 2, mitochondrial OS=Homo sapiens GN=UQCRC2 PE=1 SV=3 - [QCR2_HUMAN]	3	
Q07065	Cytoskeleton-associated protein 4 OS=Homo sapiens GN=CKAP4 PE=1 SV=2 - [CKAP4_HUMAN]	10	
Q96EP5	DAZ-associated protein 1 OS=Homo sapiens GN=DAZAP1 PE=1 SV=1 - [DAZP1_HUMAN]	3	
Q9Y295	Developmentally-regulated GTP-binding protein 1 OS=Homo sapiens GN=DRG1 PE=1 SV=1 - [DRG1_HUMAN]	4	
Q14195	Dihydropyrimidinase-related protein 3 OS=Homo sapiens GN=DPYSL3 PE=1 SV=1 - [DPYL3_HUMAN]	6	
P25685	DnaJ homolog subfamily B member 1 OS=Homo sapiens GN=DNAJB1 PE=1 SV=4 - [DNJB1_HUMAN]	3	
Q8IXB1	DnaJ homolog subfamily C member 10 OS=Homo sapiens GN=DNAJC10 PE=1 SV=2 - [DJC10_HUMAN]	11	
Q15717	ELAV-like protein 1 OS=Homo sapiens GN=ELAVL1 PE=1 SV=2 - [ELAV1_HUMAN]	8	
Q9BS26	Endoplasmic reticulum resident protein 44 OS=Homo sapiens GN=ERP44 PE=1 SV=1 - [ERP44_HUMAN]	3	
Q96AE4	Far upstream element-binding protein 1 OS=Homo sapiens GN=FUBP1 PE=1 SV=3 - [FUBP1_HUMAN]	9	
P49327	Fatty acid synthase OS=Homo sapiens GN=FASN PE=1 SV=3 - [FAS_HUMAN]	45	
Q9BZK7	F-box-like/WD repeat-containing protein TBL1XR1 OS=Homo sapiens GN=TBL1XR1 PE=1 SV=1 - [TBL1R_HUMAN]	3	
Q86UX7	Fermitin family homolog 3 OS=Homo sapiens GN=FERMT3 PE=1 SV=1 - [URP2_HUMAN]	6	
P21333	Filamin-A OS=Homo sapiens GN=FLNA PE=1 SV=4 - [FLNA_HUMAN]	44	
O75369	Filamin-B OS=Homo sapiens GN=FLNB PE=1 SV=2 - [FLNB_HUMAN]	15	
P09382	Galectin-1 OS=Homo sapiens GN=LGALS1 PE=1 SV=2 - [LEG1_HUMAN]	3	
Q08380	Galectin-3-binding protein OS=Homo sapiens GN=LGALS3BP PE=1 SV=1 - [LG3BP_HUMAN]	8	
P63244	Guanine nucleotide-binding protein subunit beta-2-like 1 OS=Homo sapiens GN=GNB2L1 PE=1 SV=3 - [GBLP_HUMAN]	12	
P07900	Heat shock protein HSP 90-alpha OS=Homo sapiens GN=HSP90AA1 PE=1 SV=5 - [HS90A_HUMAN]	9	
P08238	Heat shock protein HSP 90-beta OS=Homo sapiens GN=HSP90AB1 PE=1 SV=4 - [HS90B_HUMAN]	16	
P62805	Histone H4 OS=Homo sapiens GN=HIST1H4A PE=1 SV=2 - [H4_HUMAN]	4	

Q86X55	Histone-arginine methyltransferase CARM1 OS=Homo sapiens GN=CARM1 PE=1 SV=3 - [CARM1_HUMAN]	6	
P52292	Importin subunit alpha-1 OS=Homo sapiens GN=KPNA2 PE=1 SV=1 - [IMA1_HUMAN]	4	
Q14974	Importin subunit beta-1 OS=Homo sapiens GN=KPNB1 PE=1 SV=2 - [IMB1_HUMAN]	7	
O00410	Importin-5 OS=Homo sapiens GN=IPO5 PE=1 SV=4 - [IPO5_HUMAN]	10	
Q15181	Inorganic pyrophosphatase OS=Homo sapiens GN=PPA1 PE=1 SV=2 - [IPYR_HUMAN]	4	
P12268	Inosine-5'-monophosphate dehydrogenase 2 OS=Homo sapiens GN=IMPDH2 PE=1 SV=2 - [IMDH2_HUMAN]	9	
P35527	Keratin, type I cytoskeletal 9 OS=Homo sapiens GN=KRT9 PE=1 SV=3 - [K1C9_HUMAN]	6	
O43790	Keratin, type II cuticular Hb6 OS=Homo sapiens GN=KRT86 PE=1 SV=1 - [KRT86_HUMAN]	5	
P04264	Keratin, type II cytoskeletal 1 OS=Homo sapiens GN=KRT1 PE=1 SV=6 - [K2C1_HUMAN]	13	
P35908	Keratin, type II cytoskeletal 2 epidermal OS=Homo sapiens GN=KRT2 PE=1 SV=2 - [K22E_HUMAN]	14	
P13647	Keratin, type II cytoskeletal 5 OS=Homo sapiens GN=KRT5 PE=1 SV=3 - [K2C5_HUMAN]	7	
P08729	Keratin, type II cytoskeletal 7 OS=Homo sapiens GN=KRT7 PE=1 SV=5 - [K2C7_HUMAN]	8	
P05787	Keratin, type II cytoskeletal 8 OS=Homo sapiens GN=KRT8 PE=1 SV=7 - [K2C8_HUMAN]	7	
Q6PKG0	La-related protein 1 OS=Homo sapiens GN=LARP1 PE=1 SV=2 - [LARP1_HUMAN]	6	
P24666	Low molecular weight phosphotyrosine protein phosphatase OS=Homo sapiens GN=ACP1 PE=1 SV=3 - [PPAC_HUMAN]	3	
Q14764	Major vault protein OS=Homo sapiens GN=MVP PE=1 SV=4 - [MVP_HUMAN]	4	
P43243	Matrin-3 OS=Homo sapiens GN=MATR3 PE=1 SV=2 - [MATR3_HUMAN]	6	
O43684	Mitotic checkpoint protein BUB3 OS=Homo sapiens GN=BUB3 PE=1 SV=1 - [BUB3_HUMAN]	3	
Q9BYG3	MKI67 FHA domain-interacting nucleolar phosphoprotein OS=Homo sapiens GN=NIFK PE=1 SV=1 - [MK67I_HUMAN]	3	
Q96EN8	Molybdenum cofactor sulfurase OS=Homo sapiens GN=MOCOS PE=1 SV=2 - [MOCOS_HUMAN]	5	
O75694	Nuclear pore complex protein Nup155 OS=Homo sapiens GN=NUP155 PE=1 SV=1 - [NU155_HUMAN]	8	
P19338	Nucleolin OS=Homo sapiens GN=NCL PE=1 SV=3 - [NUCL_HUMAN]	5	

P31483	Nucleolysin TIA-1 isoform p40 OS=Homo sapiens GN=TIA1 PE=1 SV=3 - [TIA1_HUMAN]	7	
Q01085	Nucleolysin TIAR OS=Homo sapiens GN=TIAL1 PE=1 SV=1 - [TIAR_HUMAN]	6	
P51659	Peroxisomal multifunctional enzyme type 2 OS=Homo sapiens GN=HSD17B4 PE=1 SV=3 - [DHB4_HUMAN]	9	
Q8NC51	Plasminogen activator inhibitor 1 RNA-binding protein OS=Homo sapiens GN=SERBP1 PE=1 SV=2 - [PAIRB_HUMAN]	4	
Q15365	Poly(rC)-binding protein 1 OS=Homo sapiens GN=PCBP1 PE=1 SV=2 - [PCBP1_HUMAN]	7	
Q15366	Poly(rC)-binding protein 2 OS=Homo sapiens GN=PCBP2 PE=1 SV=1 - [PCBP2_HUMAN]	5	
Q8WUM4	Programmed cell death 6-interacting protein OS=Homo sapiens GN=PDCD6IP PE=1 SV=1 - [PDC6I_HUMAN]	8	
O75340	Programmed cell death protein 6 OS=Homo sapiens GN=PDCD6 PE=1 SV=1 - [PDCD6_HUMAN]	5	
Q15084	Protein disulfide-isomerase A6 OS=Homo sapiens GN=PDIA6 PE=1 SV=1 - [PDIA6_HUMAN]	6	
Q8NCA5	Protein FAM98A OS=Homo sapiens GN=FAM98A PE=1 SV=1 - [FA98A_HUMAN]	8	
Q52LJ0	Protein FAM98B OS=Homo sapiens GN=FAM98B PE=1 SV=1 - [FA98B_HUMAN]	5	
P55735	Protein SEC13 homolog OS=Homo sapiens GN=SEC13 PE=1 SV=3 - [SEC13_HUMAN]	4	
Q92734	Protein TFG OS=Homo sapiens GN=TFG PE=1 SV=2 - [TFG_HUMAN]	9	
Q13283	Ras GTPase-activating protein-binding protein 1 OS=Homo sapiens GN=G3BP1 PE=1 SV=1 - [G3BP1_HUMAN]	10	
Q9UN86	Ras GTPase-activating protein-binding protein 2 OS=Homo sapiens GN=G3BP2 PE=1 SV=2 - [G3BP2_HUMAN]	6	
O76021	Ribosomal L1 domain-containing protein 1 OS=Homo sapiens GN=RSL1D1 PE=1 SV=3 - [RL1D1_HUMAN]	4	
Q13501	Sequestosome-1 OS=Homo sapiens GN=SQSTM1 PE=1 SV=1 - [SQSTM_HUMAN]	6	
Q13242	Serine/arginine-rich splicing factor 9 OS=Homo sapiens GN=SRSF9 PE=1 SV=1 - [SRSF9_HUMAN]	5	
P67775	Serine/threonine-protein phosphatase 2A catalytic subunit alpha isoform OS=Homo sapiens GN=PPP2CA PE=1 SV=1 - [PP2AA_HUMAN]	4	
Q96HS1	Serine/threonine-protein phosphatase PGAM5, mitochondrial OS=Homo sapiens GN=PGAM5 PE=1 SV=2 - [PGAM5_HUMAN]	5	

P62140	Serine/threonine-protein phosphatase PP1-beta catalytic subunit OS=Homo sapiens GN=PPP1CB PE=1 SV=3 - [PP1B_HUMAN]	5	
Q9Y3F4	Serine-threonine kinase receptor-associated protein OS=Homo sapiens GN=STRAP PE=1 SV=1 - [STRAP_HUMAN]	7	
Q9UHB9	Signal recognition particle subunit SRP68 OS=Homo sapiens GN=SRP68 PE=1 SV=2 - [SRP68_HUMAN]	4	
P38646	Stress-70 protein, mitochondrial OS=Homo sapiens GN=HSPA9 PE=1 SV=2 - [GRP75_HUMAN]	9	
Q96GM5	SWI/SNF-related matrix-associated actin-dependent regulator of chromatin subfamily D member 1 OS=Homo sapiens GN=SMARCD1 PE=1 SV=2 - [SMRD1_HUMAN]	6	
Q92925	SWI/SNF-related matrix-associated actin-dependent regulator of chromatin subfamily D member 2 OS=Homo sapiens GN=SMARCD2 PE=1 SV=3 - [SMRD2_HUMAN]	6	
Q969G3	SWI/SNF-related matrix-associated actin-dependent regulator of chromatin subfamily E member 1 OS=Homo sapiens GN=SMARCE1 PE=1 SV=2 - [SMCE1_HUMAN]	8	
Q92804	TATA-binding protein-associated factor 2N OS=Homo sapiens GN=TAF15 PE=1 SV=1 - [RBP56_HUMAN]	7	
P17987	T-complex protein 1 subunit alpha OS=Homo sapiens GN=TCP1 PE=1 SV=1 - [TCPA_HUMAN]	11	
P78371	T-complex protein 1 subunit beta OS=Homo sapiens GN=CCT2 PE=1 SV=4 - [TCPB_HUMAN]	7	
P50991	T-complex protein 1 subunit delta OS=Homo sapiens GN=CCT4 PE=1 SV=4 - [TCPD_HUMAN]	9	
P48643	T-complex protein 1 subunit epsilon OS=Homo sapiens GN=CCT5 PE=1 SV=1 - [TCPE_HUMAN]	6	
P51532	Transcription activator BRG1 OS=Homo sapiens GN=SMARCA4 PE=1 SV=2 - [SMCA4_HUMAN]	20	
P60174	Triosephosphate isomerase OS=Homo sapiens GN=TPI1 PE=1 SV=3 - [TPIS_HUMAN]	8	
P68363	Tubulin alpha-1B chain OS=Homo sapiens GN=TUBA1B PE=1 SV=1 - [TBA1B_HUMAN]	12	
P68366	Tubulin alpha-4A chain OS=Homo sapiens GN=TUBA4A PE=1 SV=1 - [TBA4A_HUMAN]	12	
P07437	Tubulin beta chain OS=Homo sapiens GN=TUBB PE=1 SV=2 - [TBB5_HUMAN]	14	
P68371	Tubulin beta-4B chain OS=Homo sapiens GN=TUBB4B PE=1 SV=1 - [TBB4B_HUMAN]	12	
P08621	U1 small nuclear ribonucleoprotein 70 kDa OS=Homo sapiens GN=SNRNP70 PE=1 SV=2 - [RU17_HUMAN]	8	

P09012	U1 small nuclear ribonucleoprotein A OS=Homo sapiens GN=SNRPA PE=1 SV=3 - [SNRPA_HUMAN]	3	
P09936	Ubiquitin carboxyl-terminal hydrolase isozyme L1 OS=Homo sapiens GN=UCHL1 PE=1 SV=2 - [UCHL1_HUMAN]	5	
P62979	Ubiquitin-40S ribosomal protein S27a OS=Homo sapiens GN=RPS27A PE=1 SV=2 - [RS27A_HUMAN]	4	
Q14157	Ubiquitin-associated protein 2-like OS=Homo sapiens GN=UBAP2L PE=1 SV=2 - [UBP2L_HUMAN]	12	
Q12965	Unconventional myosin-Ie OS=Homo sapiens GN=MYO1E PE=1 SV=2 - [MYO1E_HUMAN]	7	
Q9Y224	UPF0568 protein C14orf166 OS=Homo sapiens GN=C14orf166 PE=1 SV=1 - [CN166_HUMAN]	7	
Q16851	UTP--glucose-1-phosphate uridylyltransferase OS=Homo sapiens GN=UGP2 PE=1 SV=5 - [UGPA_HUMAN]	3	
P08670	Vimentin OS=Homo sapiens GN=VIM PE=1 SV=4 - [VIME_HUMAN]	7	
P12956	X-ray repair cross-complementing protein 6 OS=Homo sapiens GN=XRCC6 PE=1 SV=2 - [XRCC6_HUMAN]	5	
P16989	Y-box-binding protein 3 OS=Homo sapiens GN=YBX3 PE=1 SV=4 - [YBOX3_HUMAN]	7	
P49750	YLP motif-containing protein 1 OS=Homo sapiens GN=YLPM1 PE=1 SV=3 - [YLPM1_HUMAN]	16	
Q9BYJ9	YTH domain-containing family protein 1 OS=Homo sapiens GN=YTHDF1 PE=1 SV=1 - [YTHD1_HUMAN]	4	
Q7Z739	YTH domain-containing family protein 3 OS=Homo sapiens GN=YTHDF3 PE=1 SV=1 - [YTHD3_HUMAN]	5	
Q6NZY4	Zinc finger CCHC domain-containing protein 8 OS=Homo sapiens GN=ZCCHC8 PE=1 SV=2 - [ZCHC8_HUMAN]	4	
Q92785	Zinc finger protein ubi-d4 OS=Homo sapiens GN=DPF2 PE=1 SV=2 - [REQU_HUMAN]	6	

Table S2. Mass spectrometry analysis of interacting proteins using Flag-HA-PFKP as bait

Accession	Gene	# Peptides in IgG sample	# Peptides in Flag-HA-PFKP sample	Protein Name
**Q01813**	**PFKP**		**13**	**ATP-dependent 6-phosphofructokinase, platelet type**
**Q13131**	**PRKAA1**		**5**	**5'-AMP-activated protein kinase catalytic subunit alpha-1**
Q15233	NONO		1	Non-POU domain-containing octamer-binding protein
O00571	DDX3X		1	ATP-dependent RNA helicase DDX3X
P04264	KRT1	11	11	Keratin, type II cytoskeletal 1
P13645	KRT10	12	7	Keratin, type I cytoskeletal 10
Q00610	CLTC		1	Clathrin heavy chain 1
Q92499	DDX1		1	ATP-dependent RNA helicase DDX1
Q96EP5	DAZAP1		1	DAZ-associated protein 1
P02538	KRT6A	2	1	Keratin, type II cytoskeletal 6A
P62304	SNRPE		1	Small nuclear ribonucleoprotein E
P02768	ALB	2	3	Serum albumin
P14678	SNRPB		1	Small nuclear ribonucleoprotein-associated proteins B and B'
P62318	SNRPD3		1	Small nuclear ribonucleoprotein Sm D3
